# Single-cell imaging and transcriptomic analyses of endogenous cardiomyocyte dedifferentiation and cycling

**DOI:** 10.1038/s41421-019-0095-9

**Published:** 2019-06-04

**Authors:** Yiqiang Zhang, Nuria Gago-Lopez, Ning Li, Zhenhe Zhang, Naima Alver, Yonggang Liu, Amy M. Martinson, Avin Mehri, William Robb MacLellan

**Affiliations:** 1 Division of Cardiology, Department of Medicine, University of Washington, Seattle, WA USA; 2 Center for Cardiovascular Biology, University of Washington, Seattle, WA USA; 3 Institute for Stem Cell and Regenerative Medicine, University of Washington, Seattle, WA USA; 4 State Key Laboratory for Biology of Plant Diseases and Insect Pests, Institute of Plant Protection, Chinese Academy of Agricultural Sciences, Beijing, China; 5 Department of Pathology, University of Washington, Seattle, WA USA; 6 Department of Bioengineering, University of Washington, Seattle, WA USA

**Keywords:** Mechanisms of disease, Cell growth, Cell growth

## Abstract

While it is recognized that there are low levels of new cardiomyocyte (CM) formation throughout life, the source of these new CM generates much debate. One hypothesis is that these new CMs arise from the proliferation of existing CMs potentially after dedifferentiation although direct evidence for this is lacking. Here we explore the mechanisms responsible for CM renewal in vivo using multi-reporter transgenic mouse models featuring efficient adult CM (ACM) genetic cell fate mapping and real-time cardiomyocyte lineage and dedifferentiation reporting. Our results demonstrate that non-myocytes (e.g., cardiac progenitor cells) contribute negligibly to new ACM formation at baseline or after cardiac injury. In contrast, we found a significant increase in dedifferentiated, cycling CMs in post-infarct hearts. ACM cell cycling was enhanced within the dedifferentiated CM population. Single-nucleus transcriptomic analysis demonstrated that CMs identified with dedifferentiation reporters had significant down-regulation in gene networks for cardiac hypertrophy, contractile, and electrical function, with shifts in metabolic pathways, but up-regulation in signaling pathways and gene sets for active cell cycle, proliferation, and cell survival. The results demonstrate that dedifferentiation may be an important prerequisite for CM proliferation and explain the limited but measurable cardiac myogenesis seen after myocardial infarction (MI).

## Introduction

The potential of cardiomyocytes (CMs) to proliferate is tightly developmentally controlled. The mammalian heart grows by hyperplasia during fetal life but this proliferative potential is lost in the adult. Neonatal CMs retain some proliferation capacity and can even regenerate lost myocardium after injury, but this ability is lost by 7 days after birth^1–4^. Adult CM (ACM) growth is typically hypertrophic; however, there is a very low, but measurable rate of new CM formation in adult hearts^1,5–8^. Although it has been much debated on the source of this proliferation and whether it is restricted to a subset of ACMs, the magnitude, an annual renewal rate of ~0.5–1% has now been accepted by most investigators^6,8–11^. Given the low rate of overall ACM renewal, detecting CM cell cycle progression especially cell division is challenging but crucial for future studies targeting endogenous CM regeneration. Recent strategies to quantitate new ACMs based on heavy isotope labeling appear to be more accurate than standard histology, but they require specialized expertise and equipment and are limited both in their throughput and ability to be combined with mechanistic studies. We sought to develop a simplified, high-throughput system that provides enhanced accuracy and facilitates the study of the cellular sources and mechanisms underlying CM renewal in adult mice.

Theoretically, new CMs could arise from the differentiation of resident cardiac progenitor cells (CPCs) or by the proliferation of pre-existing CMs^12,13^. CPCs are essential for normal cardiac development, but their role, if any, in adult hearts is disputed and uncertain^4,14–24^. Recent genetic cell fate tracking and clonal analyses demonstrated that new myocytes likely arise from pre-existing CMs. However, incomplete genetic labeling of ACMs in previous Cre/LoxP models and inefficient gene recombination inherent in “mosaic analysis with double markers” model have made the findings inconclusive^5,8^. In order to fully characterize endogenous myocyte renewal, we developed a new bi-transgenic αMHC-MCM;RFP^fl^/GFP system that has improved CM fate mapping^25,26^.

Dedifferentiation is a regressive process where specialized cells or tissues regain primitive phenotypes—this is critical for repair and regeneration in many lower vertebrates. While ACMs are known to be able to dedifferentiate and re-differentiate both in cell culture and when transplanted into post-infarct myocardium^27–31^, whether this happens in vivo and how to accurately quantify the magnitude of myocyte dedifferentiation is unknown. To address these limitations, we created a cardiac nucleus-specific reporter transgenic mouse *Tg(Myh6-H2BBFP6xHis)* referred to as the blue fluorescent protein (BFP) model. This BFP mouse model enabled the high-throughput quantification of ACMs and their dedifferentiation. BFP signal was highly expressed in ACMs but reduced in dedifferentiated ACMs and immature myocytes, such as those from neonatal hearts. When our BFP mice are bred to bi-transgenic αMHC-MCM;RFP^fl^/GFP mice, the new triple transgenic αMHC-MCM;RFP^fl^/GFP;BFP mice provide a genetic model to visualize and quantitate dedifferentiated CMs in vivo^5,8,12,18,19,21,29–33^. Using these novel transgenic models, we demonstrated that CM dedifferentiation occurs after cardiac injuries, and is associated with the enhanced ACM cycling in post-infarct hearts. Massive parallel single-nucleus RNA-seq (snRNA-seq) analysis revealed novel transcriptomes in the subset of CMs expressing specific transgene reporters consistent with dedifferentiation and active cell cycling. This model provides a useful tool to study the mechanisms controlling endogenous myocardial regeneration in injured hearts by combining high-throughput single-cell imaging and transcriptomic analyses.

## Results

### Minimal contribution of non-myocyte pools to cardiomyocyte renewal in post-infarct hearts

**Fig. 1:**
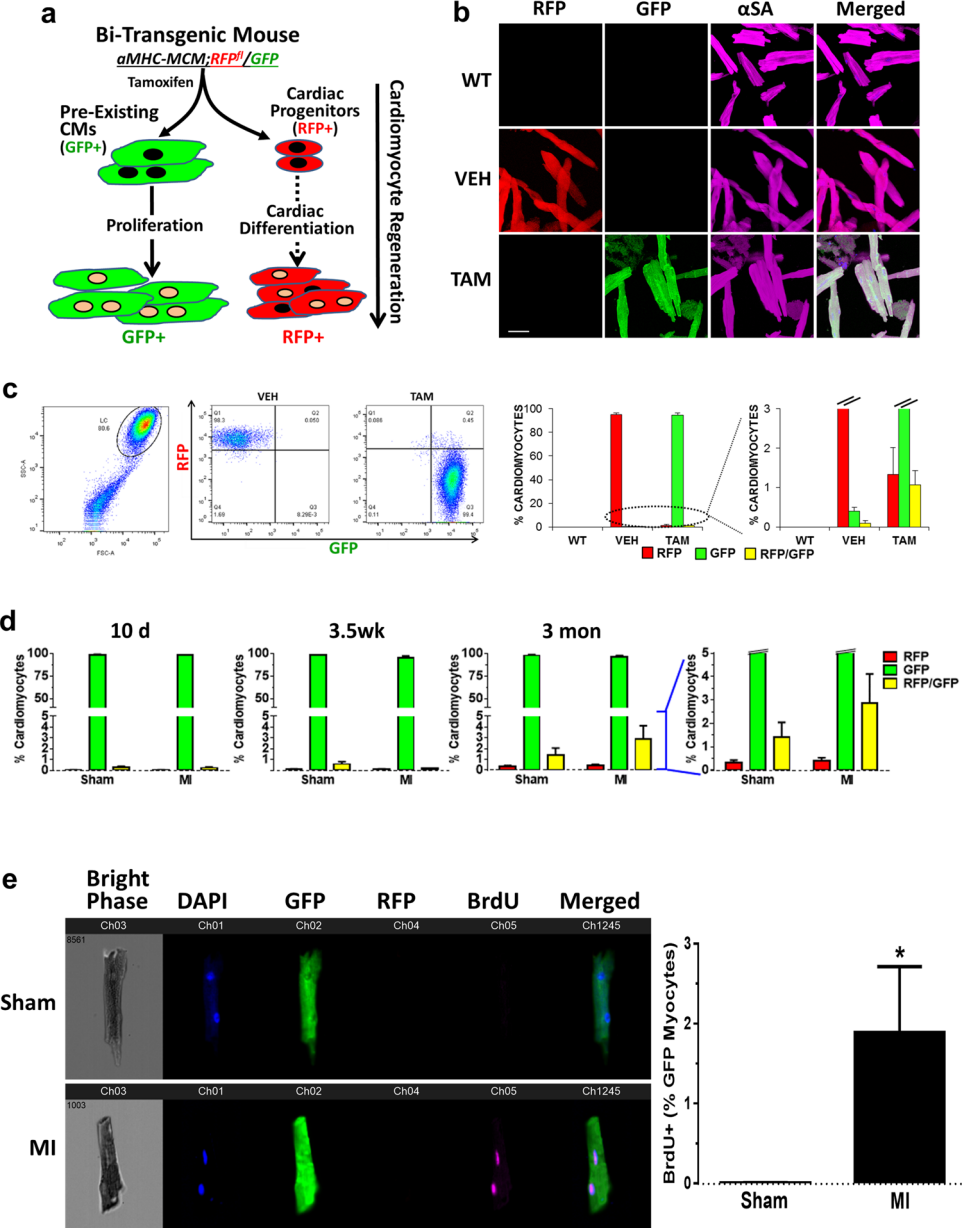
Non-cardiomyocyte pools do not contribute to CM renewal in post-infarct hearts of bi-transgenic mice. **a** Cardiomyocyte renewal can potentially originate from pre-existing cardiomyocytes (GFP^+^) or resident progenitors (RFP^+^) in tamoxifen-treated bi-transgenic αMHC-MCM;RFP^fl^/GFP mice. **b** α-sarcomeric actinin (αSA, magenta) immunostaining on myocytes isolated from bi-transgenic αMHC-MCM;RFP^fl^/GFP mice with tamoxifen (TAM) or vehicle (VEH) treatment, or cells from wild-type (WT) littermates. Scale bar = 50 µm. **c** Flow cytometry analysis showing the expression of GFP and RFP in myocytes isolated from hearts of bi-transgenic mice without (VEH) or with tamoxifen (TAM) treatment. The far-left panel shows the total ventricular populations containing small cells (non-myocytes), and larger cells (circled) that were either RFP^+^ (VEH; 2nd dot plot) or GFP^+^ (TAM; 3rd dot plot). *n* = 3 mice for each group. **d** Expression profile of GFP and RFP in cardiomyocytes from bi-transgenic mouse ventricles 10 days, 3.5 weeks, or 3 months after MI or Sham operation. Statistics: *p* > 0.05 in two-way ANOVA analysis (*n* = 3–4 mice for each group). **e** ImageStream analysis on total ventricle cells from 3.5-week post-MI or sham mice. GFP and RFP are shown in channel Ch02 and Ch04, respectively; and BrdU incorporation signal revealed by Alexa Fluor 647-conjugated antibody is shown in Ch05; nuclear staining (by DAPI) in Ch01; and the bright phase signal in Ch03. *n* = 3 mice (Sham or MI). *p < 0.05 in *t*-test

To determine the potential contribution of non-myocyte populations, including putative resident CPCs to CM renewal^1,5,8,18,19,21,29,31^, we generated a bi-transgenic αMHC-MCM;RFP^fl^/GFP mouse model by cross-breeding αMHC-MCM mouse with Rosa26-mT/mG reporter mouse (the latter referred to as RFP^fl^/GFP mouse for its dual-color reporters in red and green fluorescences) (Fig. 1a; and Supplementary Fig. S1a)^25,26^. This bi-transgenic mouse model has a tamoxifen-inducible, CM-specific GFP signal that is superior to previously reported systems^8,25,26,29^. More than 98% of CMs in tamoxifen-treated bi-transgenic adult mice irreversibly switched from RFP to GFP expression (Fig. 1b, c). Immunostaining revealed the co-expression of GFP signal with CM markers, such as α-myosin heavy chain (αMHC), Tropomyosin, α-sarcomeric actinin (α-SA), and troponin I (cTnI) (Supplementary Fig. S1b, c). After tamoxifen treatment, over 98% ACMs were GFP^+^; and there was a minor portion of CMs that co-expressed GFP and RFP (<1%), likely due to asymmetric gene recombination^34^ in ACMs that were binucleated (Fig. 1c). There was a barely detectable GFP^+^ population (<0.03%) among small non-myocytes, confirming that gene recombination was specific to ACMs, without leaky labeling of putative non-myocytes, such as CPCs or committed cardiac precursors. Therefore, the GFP population specifically identified the vast majority of pre-existing ACMs in tamoxifen-treated bi-transgenic mice.

To determine if cardiac differentiation of any putative resident CPCs among the non-myocyte pool contributed to ACM renewal in post-infarct hearts, we induced myocardial infarction (MI) in tamoxifen-treated αMHC-MCM;RFP^fl^/GFP adult mice and followed them for the appearance of “new” RFP-positive cardiac myocytes. There was no significant change in the percentage of GFP^+^ cells among CMs up to 3 months after MI compared to sham-operated hearts (96.5% in MI versus 97.9% in Sham) (Fig. 1d). The amount of RFP^+^ CMs remained minimal, and showed no significant difference between sham and post-infarct hearts. To track ACM cell cycle activity, we labeled cycling cells in sham or post-MI bi-transgenic mice for 3.5 weeks via drinking water containing 5-bromo-2'-deoxyuridine (BrdU). BrdU^+^ GFP ACMs were significantly increased in post-infarct hearts compared to sham hearts as revealed by ImageStream, a multispectral, microscopy imaging-based flow cytometry analysis (Fig. 1e). No RFP^+^BrdU^+^ ACMs were detected in either post-MI or sham hearts (data not shown). These results are consistent with the notion that non-cardiomyocyte pools do not contribute to ACM renewal in post-infarct hearts and suggest that if new myocytes are formed they arise from pre-existing CMs^8^.

### Generation of a cardiac-specific nuclear BFP reporter mouse to visualize myocyte maturity and dedifferentiation

**Fig. 2:**
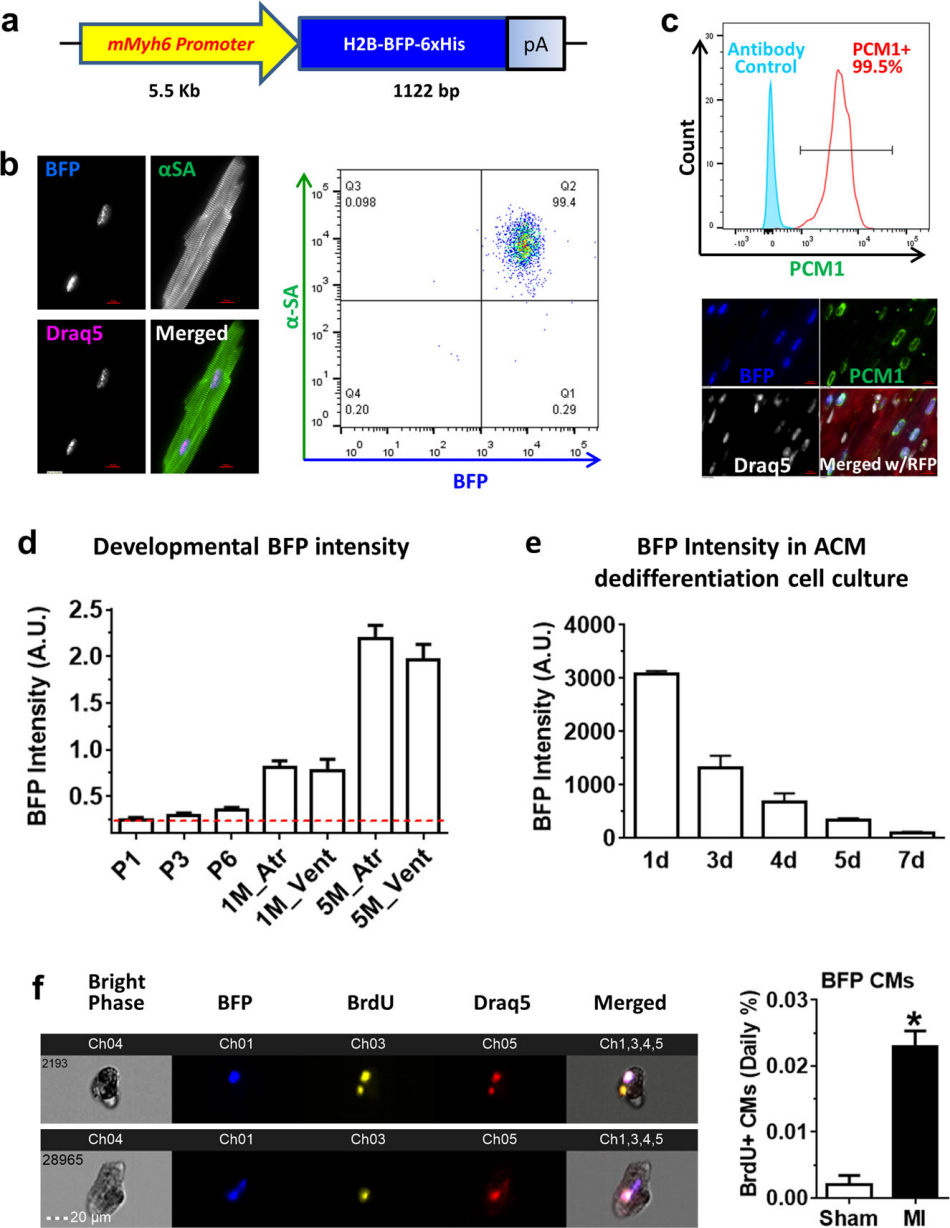
Creation of cardiomyocyte nucleus-specific transgenic BFP mice. **a** Gene construct for transgenic cardiac nuclear blue fluorescent protein (BFP) reporter mouse. A full-length mouse α-MHC (*mMyh6*) promoter drives an in-frame fusion gene containing *H2B*, *BFP*, and a tag of six histidines (6xHis). Scale bar = 10 μm. **b** Confocal images (left panels) and flow cytometry dot plot (right panel) showing cardiomyocyte nucleus-specificity of BFP signal. Native blue fluorescent signal from BFP transgene was found in cardiomyocytes identified by α-sarcomeric actinin (α-SA, green); nuclei were stained by Draq5 (magenta). Scale bar = 10 μm. For flow cytometry analysis, samples were gated on Draq5^+^ population and α-SA (Alexa Fluor 488) and native BFP signal was analyzed. **c** Upper panel: Flow cytometry histogram showing the expression of pericentriolar material 1 (PCM1) (Alexa Fluor 488) in BFP^+^ population in the total heart nuclei preparations. 98.8±0.9% BFP^+^ nuclei are PCM1^+^ (*n* = 3 mice). Lower panel: Confocal image showing the expression of PCM1 (green) and native BFP signal (blue) in ventricular tissue of transgenic mouse carrying BFP and Rosa26-mT/mG (RFP^fl^/GFP) transgenes. RFP expressed ubiquitously in all cells. Nuclei were stained with Draq5 (pseudo-white). Scale bar = 100 µm. **d** Native BFP signal intensity normalized to DNA content determined by Draq5 staining in heart tissues. *p* < 0.0001 with one-way ANOVA test. *n* = 3, 4, 3, 3, and 4 mice for P1, P3, P6, 1 month, and 5 months ages, respectively. **e** BFP signal determined by live cell imaging during adult CM dedifferentiation in cell culture. *p* < 0.0001 with one-way ANOVA test. *N* = 300–500 cells from three cell cultures. **f** ImageStream analysis of total ventricular cell preparations. Left panels: example BrdU^+^ BFP cardiomyocytes. BFP signal is shown in Ch01, BrdU incorporation signal in Ch03, nuclear staining (by Draq5) in Ch05, and the bright phase signal in Ch04. *p < 0.01 in *t*-test (*n* = 3 mice; Sham or MI)

To track CMs with a real-time reporter of CM maturity, we generated an ACM nucleus-specific transgenic BFP mouse model. The *BFP* gene was fused in-frame to histone *H2B* gene under the control of cardiac-specific αMHC (*Myh6*) promoter. Therefore, only ACMs expressed BFP (Fig. 2a, b; and Supplementary Figs. S2 and S3). BFP^+^ nuclei also demonstrated strong co-expression of pericentriolar material 1 (PCM1) which has been used to identify ACM nuclei previously (Fig. 2c)^11^. Transgene expression did not adversely affect cardiac development or growth, and transgenic mice had normal cardiac histology and function (Supplementary Fig. S4). Approximately 35% of total ventricular nuclei were BFP^+^ when assayed by either imaging native BFP fluorescent signal, or by immune reactivity to BFP and polyhistidine-tag proteins (Supplementary Fig. S5). BFP signal was expressed in over 99% ACMs identified by the expression of α-SA or cardiac Troponin T (cTnT) (Fig. 2b; and Supplementary Figs. S2, S3, and S5). Analyses of a panel of major organs further confirmed the expression of BFP was specific to CMs (Supplementary Figs. S6 and S7). Comparable BFP^+^ populations were seen in the ventricles of young (1 month old) and adult (5 months old) hearts (Supplementary Fig. S8). Importantly, the BFP signal was developmentally regulated: minimally expressed in early neonatal CMs, but significantly augmented in ACMs (Fig. 2d; and Supplementary Fig. S9a and b). When isolated BFP ACMs were subjected to dedifferentiation cell culture conditions, BFP signal reduced rapidly within a week (Fig. 2e; and Supplementary Fig. S9c), consistent with the reduced αMHC expression in dedifferentiated CMs^29^. Therefore, BFP signal could potentially be used as a surrogate to visualize CM maturity, and BFP signal reduction in ACMs may reflect their dedifferentiation.

To quantify ACM cycling in this model, we treated sham-operated or post-MI transgenic BFP mice with BrdU for 3.5 weeks. Approximately 30% BFP^+^ myocytes were lost in post-infarct hearts compared to sham as evaluated by ventricular BFP^+^ nuclei (Supplementary Fig. S10). In surviving myocytes, there was a significant increase (9.2-fold) of BFP^+^BrdU^+^ cells in post-MI hearts compared to sham mice (Fig. 2f). This labeling rate was comparable to previous isotope labeling studies when converted to similar pulsing period: 0.47% of all CMs in the whole-ventricle in our single-cell imaging analysis versus 1.2% specifically in the infarct border zone measured with multi-isotope imaging mass spectrometry (MIMS) analysis^8^.

### Increased dedifferentiated ACMs in post-infarct multi-reporter tri-transgenic mice

**Fig. 3:**
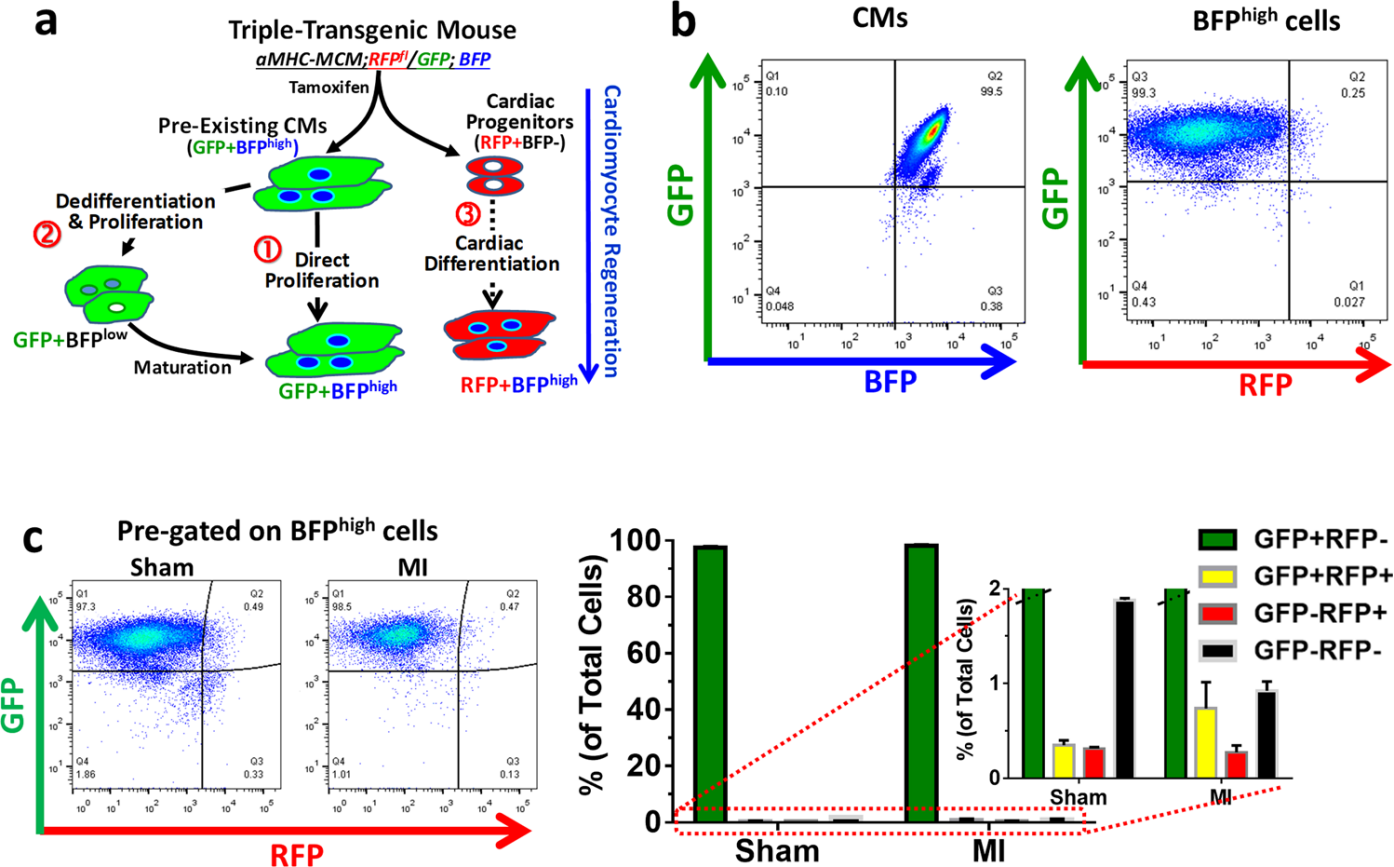
Adult cardiomyocyte renewal in post-infarct tri-transgenic mouse hearts. **a** Potential cellular mechanisms underlying endogenous CM renewal in the triple-transgenic mouse model. **b** Flow cytometry dot plot showing specific expression of both genetic GFP tag and BFP reporter in CM from mice after tamoxifen-induced gene recombination. Nuclei were stained with Draq5. The shown cell dot plot is pre-gated on BFP^high^ population. **c** Characterization of cell populations in tri-transgenic mouse hearts 1.5 weeks after Sham or myocardial infarction (MI). Nuclei were stained with Draq5. No significant changes in BFP myocytes with GFP or RFP reporter. *n* = 3 mice (Sham or MI)

Next, we sought to identify ACM dedifferentiation in vivo by creating a tri-reporter mouse model. By crossbreeding the mouse model for permanent GFP labeling in CMs (αMHC-MCM;RFP^fl^/GFP model) with the mice that express a CM-specific, maturity/dedifferentiation reporter (BFP model), we created a triple-transgenic mouse model. In this model, dedifferentiation of pre-existing ACMs would be identified as GFP^+^BFP^low^ (or GFP^+^BFP^−^) cells (Fig. 3a).

**Fig. 4:**
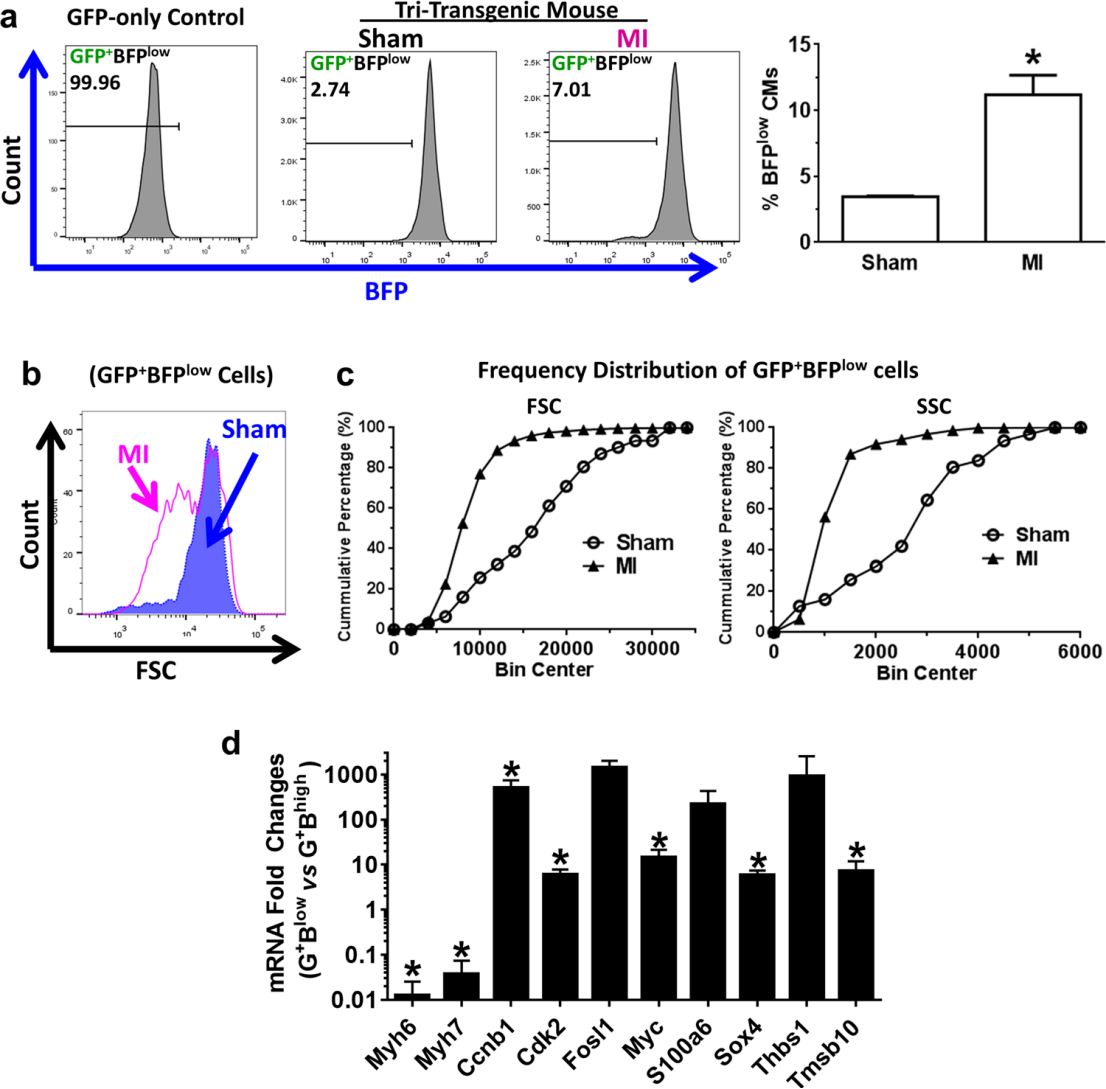
Increased dedifferentiation GFP myocytes in post-infarct tri-transgenic mouse hearts. **a** Left panels: flow cytometry histogram. GFP-only control heart cells were used to set the gate. Bar chart*:* Percentage of BFP^low^ cells among GFP^+^ myocytes in ventricles of 1.5-week post-MI or Sham hearts. *n* = 3 mice, *t*-test. *p < 0.05. **b** Representative forward side scatter (FSC) histograms of gated GFP^+^BFP^low^ populations in post-MI and Sham hearts. **c** Frequency distribution of FSC and side scatter (SSC) indices of GFP^+^BFP^low^ cells from post-MI or Sham hearts (*n* = 3 mice). One-way ANOVA test: *p* < 0.0001 for both FSC and SSC indices, comparing MI to Sham. **d** RT-qPCR analysis of gene expression in GFP^+^BFP^−^ (G^+^B^low^) myocytes compared to GFP^+^BFP^+^ (G^+^B^high^) myocytes isolated from post-infarct tri-transgenic mouse hearts. *p < 0.05, G^+^B^low^ vs. G^+^B^high^ (*n* = 3 mice)

After tamoxifen treatment, over 99% GFP^+^ CMs were BFP^+^ in tri-transgenic mice, and RFP^+^ BFP^+^ cells were minimal among ACMs (Fig. 3b). After MI, there was no significant change in GFP and RFP expression among BFP^+^ ACMs when compared to those in sham-operated hearts, consistent with our previous results (Fig. 3c). In total ventricular cells, only ~0.02% were RFP^+^BFP^+^ at baseline, which might represent the minimal RFP^+^ ACMs that had failed to undergo Cre/LoxP gene recombination. The RFP^+^BFP^+^ population remained minimal and not different between post-MI or sham hearts (Fig. 3c). Although ACMs suggestive of dedifferentiation have been shown in previous studies, definitive in vivo proof has been lacking. As shown in Fig. 4a, there was approximately a three-fold increase in the rare GFP^+^BFP^low^ population in post-MI hearts compared to sham hearts. The forward and side scatter indices (FSC and SSC, respectively) for GFP^+^BFP^low^ cells in post-MI hearts were significantly lower than those in sham-operated hearts (Fig. 4b, c), suggesting they were smaller and had rounder morphology.

Definitively identifying dedifferentiated CM is problematic, as no consensus exists on the specific molecular signatures. To identify genes that were unique to the dedifferentiated state, we performed whole-transcriptome analysis on in vitro dedifferentiated CM, embryonic CMs, normal ACMs or hypertrophic ACMs. Dedifferentiated CMs displayed a distinct transcriptional profile (Supplementary Fig. S11). Dedifferentiation-specific genes were defined as those upregulated in dedifferentiated CMs compared to normal ACMs, excluding genes that were more highly expressed in embryonic CMs or hypertrophic ACMs (Supplementary Table S1). We then used this molecular signature to compare GFP^+^BFP^high^ (normal ACMs) to GFP^+^BFP^low^ cells in post-infarct hearts. Consistent with in vitro results, GFP^+^BFP^low^ cells expressed significantly higher levels of these dedifferentiation genes, including Fos-like antigen 1 (*Fosl1*), myelocytomatosis oncogene (*Myc*), S100 calcium-binding protein A6 (*S100a6*), SRY-box containing gene 4 (*Sox4*), and thymosin beta 10 (*Tmsb10*). In addition, cardiac marker *Myh7* was significantly higher than *Myh6* in GFP^+^BFP^low^ myocytes although both transcripts were down-regulated (Fig. 4d). Thus, GFP^+^BFP^low^ cells in tri-transgenic hearts are morphologically and molecularly similar to dedifferentiated CM^27,29–31^.

### Dedifferentiated CMs contributed to active cycling ACMs in post-infarct hearts

**Fig. 5:**
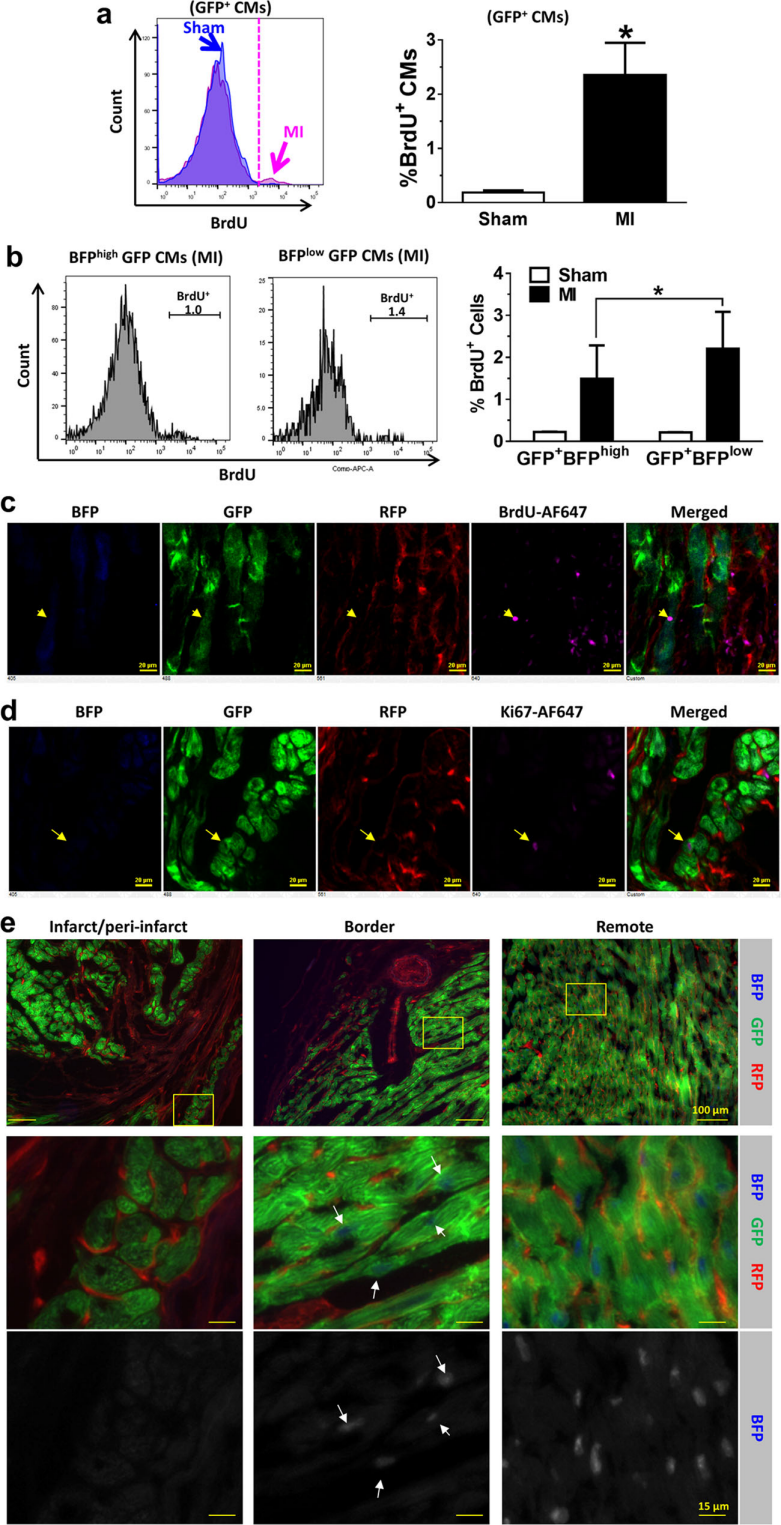
Increased cell cycling in dedifferentiated GFP^+^ cardiomyocytes in post-infarct hearts. **a** and **b** Percentages of BrdU^+^ CMs in (**a**) mature myocytes (GFP^+^BFP^high^) or (**b**) dedifferentiated myocytes without BFP expression (GFP^+^BFP^low^). *p < 0.05. *n* = 3 mice (Sham or MI). **c**, **d** BrdU incorporation (**c**) and Ki67 expression (**d**) in post-infarct ventricles. Arrows denote the BrdU or Ki67 positive GFP^+^ CM. Scale bar = 20 µm. **e** Changes in BFP signal in infarct/peri-infarct area, infarct border zone, and remote regions. Boxed regions are shown in middle and lower panels. White arrows show weaker BFP signal in GFP^+^ cardiomyocytes in the infarct border zone. Scale bar = 100 µm for the first row, and 15 µm for other rows

Dedifferentiated ACMs in tissue culture demonstrate increased cell cycling and are capable of proliferation^29,30^. Given the increased cycling of GFP myocytes in post-MI bi-transgenic mice (Fig. 1e) and that GFP^+^BFP^low^ cells expressed higher cell cycle genes, such as *Ccnb1* and *Cdk2* (Fig. 4d), we hypothesized that BFP^low^ ACMs might have enhanced cell cycling and proliferation activity. Flow cytometry analysis revealed similar levels of BrdU^+^ GFP CMs in post-MI hearts of tri-transgenic mice compared to post-MI bi-transgenic hearts, which were both significantly higher than that in sham-operated hearts (Fig. 5a). However, the rate of cycling was ~50% higher in the dedifferentiated ACM subpopulation (GFP^+^BFP^low^; 2.2%) compared to normal, mature ACMs (GFP^+^BFP^high^; 1.49%) (Fig. 5b). To identify regional differences in CMs dedifferentiation and cell cycle activity in post-infarct hearts, we performed high-content imaging analysis of whole-ventricular sections (Fig. 5c, d). The BFP signal in cycling GFP^+^ myocytes was barely detectable in peri-infarct zones, with reduced expression in border zones, while expression remained at normal levels in remote areas similar to that in sham-operated hearts (Fig. 5e). These results suggest that CM dedifferentiation and cell cycle activity might be regulated locally in response to post-infarct remodeling of the myocardium^35–37^.

**Fig. 6:**
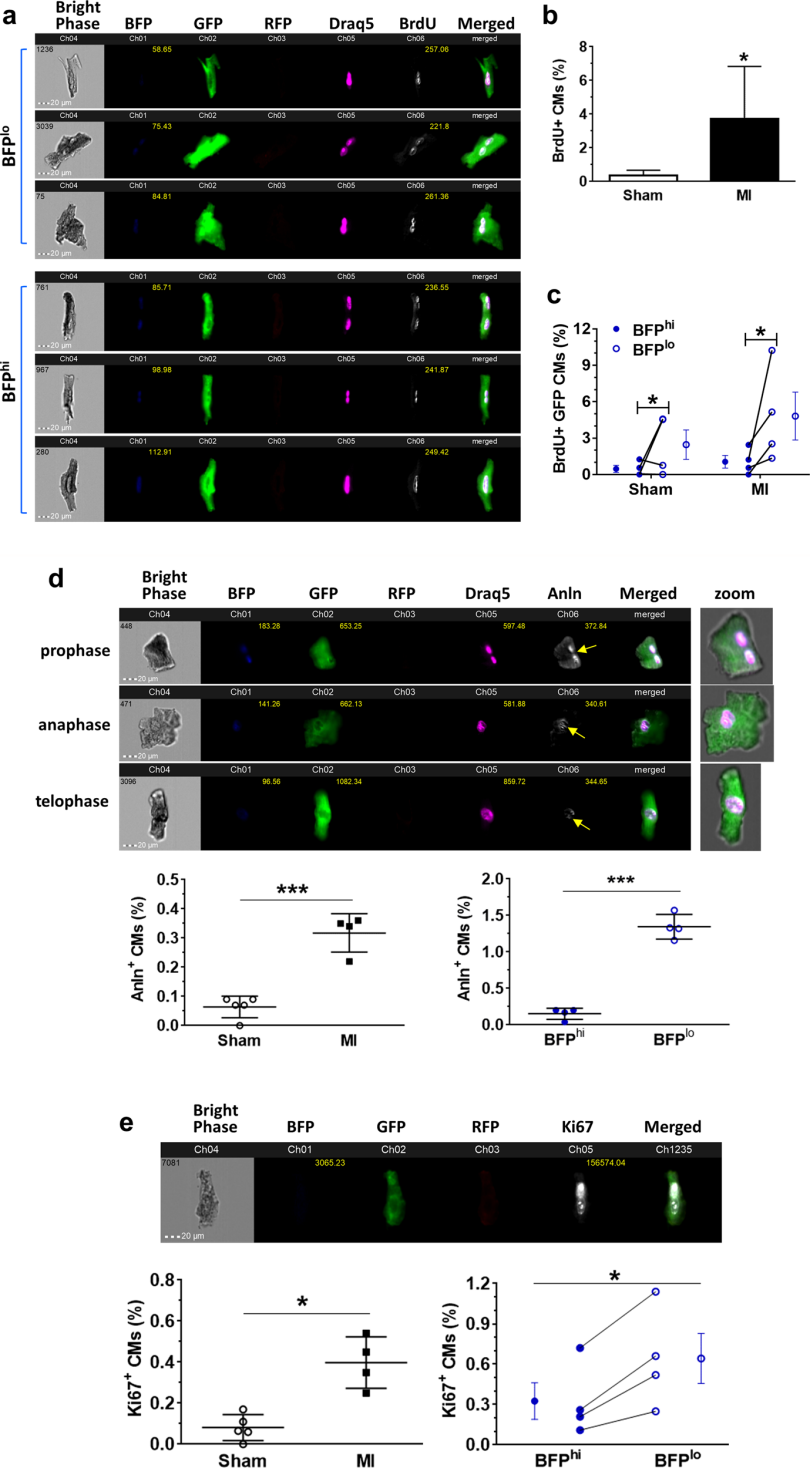
Dedifferentiated cardiomyocytes (GFP^+^BFP^low^) demonstrated higher rates of cell cycling. **a** ImageStream analysis of ventricular cells from tri-transgenic mice. Images demonstrate GFP^+^BrdU^+^ myocytes in post-infarct hearts, with BFP and BrdU signals indicated by median pixel intensity. Nuclear BFP signals were classified as no or low (BFP^low^) and high (BFP^high^) (see “Materials and methods” for details). **b** BrdU^+^ GFP CM in post-infarct (MI) or sham hearts. *p < 0.05 by *t*-test. **c** BrdU^+^ GFP CM in a population with high or low BFP signal (BFP^high^ and BFP^low^, respectively). *p* > 0.05 by two-way ANOVA test. *p < 0.05 by post-hoc Bonferroni test. Connected line represents each mouse. **d** ImageStream analysis of anillin (Anln)^+^ CM. Nuclei (stained by Draq5) and Anln (revealed by APC-Cy7-conjugated antibody) are shown in Ch05 and Ch06, respectively. Yellow arrows denote the mid-body plane. Lower panels show the percentage of Anln^+^ GFP CM in post-MI or sham hearts (left), and in BFP^high^ or BFP^low^ GFP CM subpopulations from post-infarct hearts (right). *p < 0.001 by *t*-test. **e** ImageStream analysis of Ki67^+^ CM. Lower panels show the percentage of Ki67^+^ GFP cardiomyocytes in post-infarct or sham hearts (left), and in the BFP^high^ or BFP^low^ GFP CM subpopulations from post-infarct hearts (right). *p < 0.05 by *t*-test. Paired post-infarct hearts identified by connected lines

To assess overall CM dedifferentiation and cell cycle activities in the whole heart, we examined individual heart cells from tri-transgenic mice using single-cell ImageStream analysis (Fig. 6a). There was a significant increase (10.8-fold) of BrdU^+^ GFP myocytes in post-MI hearts compared to sham hearts (Fig. 6b). The GFP^+^BFP^low^ ACM population had a significantly higher BrdU^+^ incorporation rate compared to GFP^+^BFP^high^ ACMs, indicating a higher rate of cycling in dedifferentiated (GFP^+^BFP^low^) ACMs (Fig. 6c). To determine if this cycling progressed to cytokinesis we examined the expression of Anillin (Anln) and found that there were more Anln^+^ cells among the BFP^low^ GFP ACMs compared to that in BFP^high^ GFP ACM population (Fig. 6d). Cytoskeletal structure in cycling CM appeared to be less organized consistent with a dedifferentiated cell state (Supplementary Fig. S12). ACMs cycling estimated by Ki67 expression was 4.6-fold higher in post-infarct hearts compared to sham hearts; and there was a ~2-fold increase of Ki67^+^ cells in BFP^low^ CMs compared to BFP^high^ CMs (0.63% versus 0.34%) (Fig. 6e). In contrast to Ki67^−^ CMs in normal hearts being mainly binucleated, cycling (Ki67^+^) CMs from post-MI hearts were predominately mononucleated (Supplementary Fig. S13). These data suggest that dedifferentiated (BFP^low^) ACMs are more actively cycling and may be more likely to divide.

### Transcriptomic analysis of in vivo cardiomyocyte dedifferentiation

**Fig. 7:**
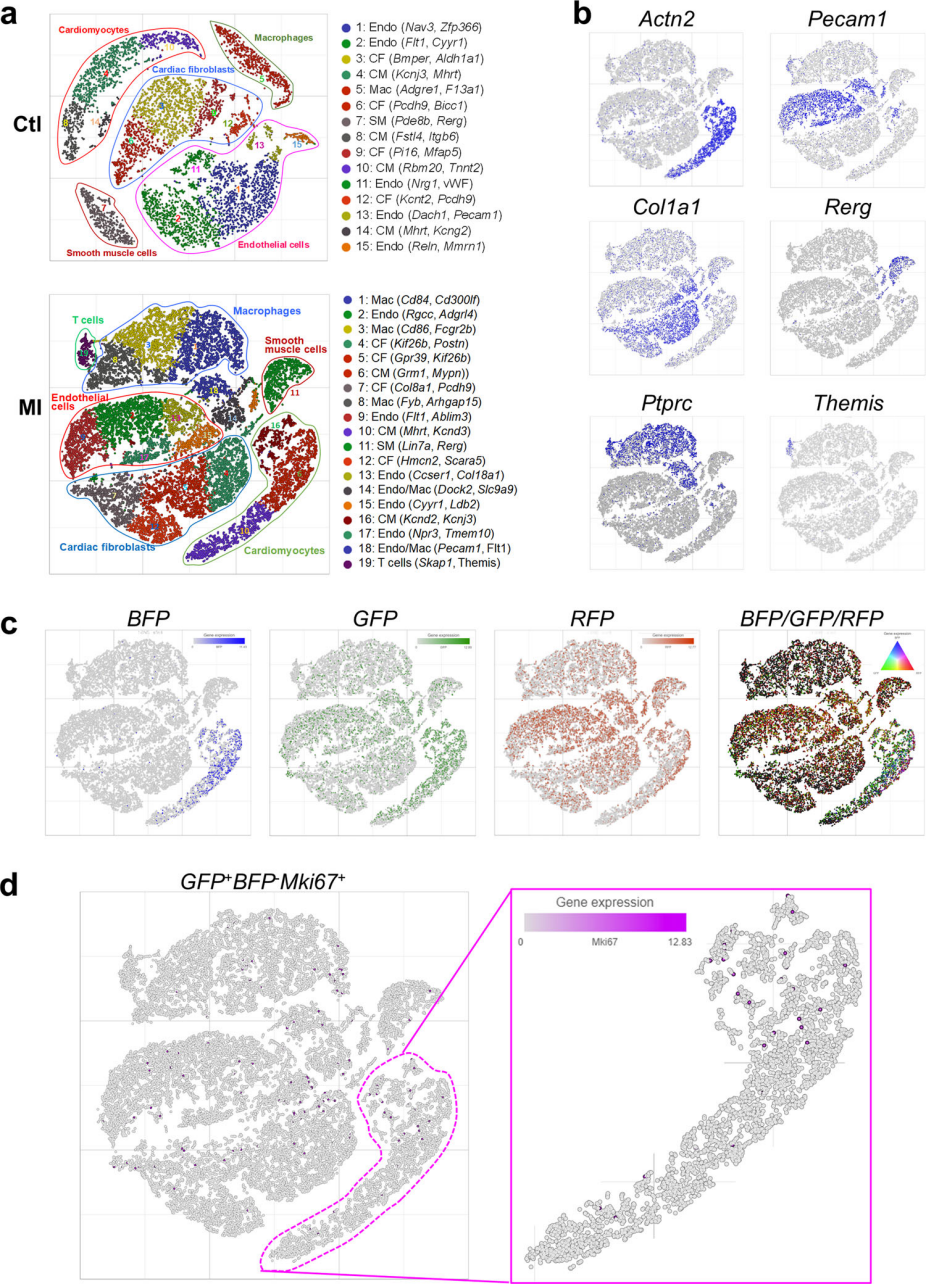
Single-nucleus RNA-seq reveals specific cell populations in the heart. **a** t-SNE plots showing graph clustering of heart cells in normal control (Ctl) and post-infarct (MI) hearts. Top-ranked genes differentially expressed in clusters are bracketed. CM cardiomyocytes, CF cardiac fibroblasts, Endo endothelial cells, SM smooth muscle cells, Mac macrophages. **b** Expression levels of positive expression level (low-gray, high-blue) of markers in myocytes (*Actn2*), endothelial cells (*Pecam1*; Cd31), cardiac fibroblasts (*Col1a1*), smooth muscle cells (*Rerg*), and macrophages *(Ptprc*; Cd45), and T cells (*Themis*) in post-MI hearts. **c** Expression of *BFP*, *GFP*, and *RFP* reporter genes in nuclei of post-infarct hearts. **d** The t-SNE plot for nuclei from post-infarct hearts showing dedifferentiated cycling CM expressing *GFP*^+^*BFP*^−^ *Mki67*^+^ (circled nuclei; *Mki67* expression level: low-gray, high-blue). There were 165 Ki67^+^ nuclei among 1939 *BFP*^−^ ACM nuclei

To dissect transcriptomic reprogramming of dedifferentiated myocytes in post-infarct hearts, we performed massive parallel single-nucleus RNA-sequencing (snRNA-seq) using a modified 10x Genomics protocol allowing a targeted analysis of the highly heterogeneous cell populations in the heart. After filtering and data normalization, we obtained high-quality single-nucleus datasets for 22,992 nuclei from post-MI hearts, and 8550 nuclei for control myocardium. Unsupervised graph-based clustering with smart local moving (SLM) algorithm revealed 15 clusters of cells in the control (Fig. 7a), including common cardiac populations: CMs expressing structural genes (*Actn2*, *Myl2*, *Tnnt2*, *Tpm1*) and ion channel genes (*Scn5a*, *Kcnj3*, and *Kcnd2)*; cardiac fibroblasts expressing *Col3a1* and *Ddr2*; endothelial cells expressing *Pecam1* (Cd31) and *Tie1*; smooth muscle cells expressing *Mylk, Pde8b, and Rerg*; and a small fraction of macrophages (Fig. 7a; Supplementary Fig. S14a, and Supplementary Table S2). In post-infarct hearts, the myocyte nuclei population was reduced, and inflammatory cells such as macrophages (*CD45*, *Ccr5*) and B/T cells (*CD74*, *Fcgr2b*, *Themis*) increased (Fig. 7a, b; and Supplementary Table S3 and S4). *BFP* and *GFP* transcripts were enriched in the putative CM clusters in both control and post-infarct hearts. The *BFP*^−^/*BFP*^+^ ratio of CM populations (*GFP*^+^) increased from 0.93 (444/475) in the control to 9.3 (1939/209) in post-MI hearts (Fig. 7c, and Supplementary Fig. S14b) similar to findings from the flow cytometry analysis (Fig. 4a). While *Mki67* was barely detectable in ACM nuclei from the control, there was a significant increase of nuclei expressing *Mki67* in the *GFP*^+^*BFP*^−^ population in post-MI hearts (Fig. 7d, and Supplementary Fig. S15).

**Fig. 8:**
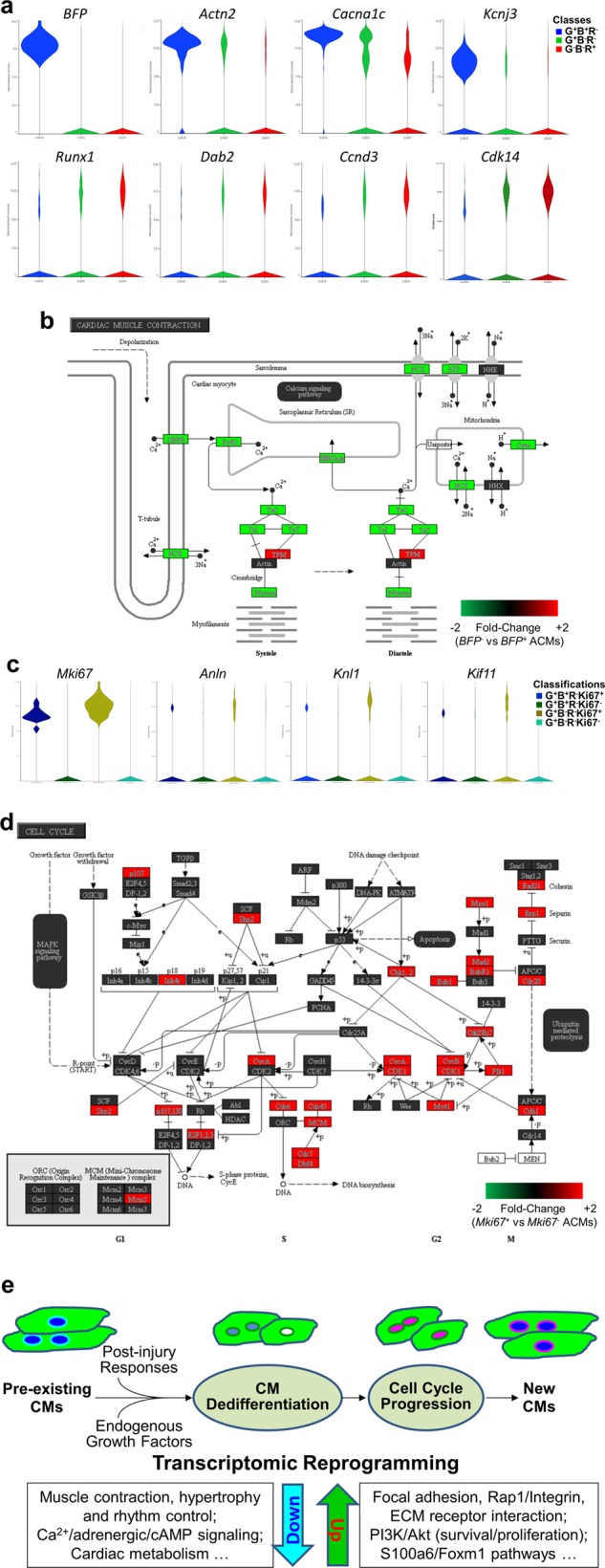
Cardiomyocyte dedifferentiation and cell cycling revealed by single-nucleus transcriptomes. **a** Violin plots showing the expression of genes in *GFP*^+^*BFP*^+^*RFP*^−^ (G^+^B^+^R^−^; *blue*), *GFP*^+^*BFP*^−^*RFP*^−^ (G^+^B^−^R^−^; *green*), and *GFP*^−^*BFP*^−^*RFP*^+^ (G^−^B^−^R^+^; *red*) nuclei from post-infarct hearts. Normalized expression values for these genes had a |Fold-change| > 2 with a FDR, *p* ≤ 0.01. **b** Enrichment of *Cardiac muscle contraction* pathway (enrichment score 10.64, p 2.39E−05) by the DEGs (|Fold-change| > 2; FDR, *p* ≤ 0.01) between *BFP*^−^ and *BFP*^+^ CM nuclei (*GFP*^+^*RFP*^−^) from post-infarct hearts. **c** Expression of cell cycle genes in normal or dedifferentiated cardiomyocytes nuclei from post-infarct hearts. **d** Enrichment of *cell cycle* pathway (enrichment score 36.974; p 8.76E−17) by the DEGs (|fold-change| > 2; FDR *p* ≤ 0.01) between *Mki67*^+^ and *Mki67*^−^ dedifferentiated cardiomyocyte nuclei (*GFP*^+^*BFP*^−^*RFP*^−^) from post-infarct hearts. **e** Working model of CM regeneration in vivo

In the *GFP*^+^ ACM population, there were 2595 and 1499 DEGs between *BFP*^−^ and *BFP*^+^ nuclei in control and post-infarct hearts, respectively (Supplementary Fig. S16). Compared to *BFP*^+^ nuclei, *BFP*^−^ expressed significantly lower levels of cardiac genes (e.g. *Actn2*, *Cacna1c*, *Kcnj3*, *Myh6*, *Scn5a*, *Tnnt2*), with increases in genes associated with dedifferentiation (*Runx1* and *Dab2*; Fig. 8a)^27,31^. The genes upregulated during ACM in vitro dedifferentiation (Fig. 4d) were also increased in *BFP*^−^ ACM nuclei compared to *BFP*^+^ ones, for example: *S100a6* (5.37-fold; *p* = 0.000355), *Tmsb10* (3.81-fold; *p* = 0.000786), *Thbs1* (3.45-fold, *p* = 0.0594). *BFP*^−^ CM nuclei also expressed higher levels of active cell cycle genes, such as *Ccnd3*, *Cdk14* (Fig. 8a). KEGG pathway enrichment analysis on DEGs in *BFP*^−^ (versus *BFP*^*+*^) ACMs from post-infarct hearts revealed a number of affected pathways with reduced gene expression, such as those in pathways for cardiac muscle contractile function (Fig. 8b), hypertrophic remodeling, and calcium, adrenergic, and cAMP signaling pathways, and cardiac rhythm (Supplementary Fig. S17a–e; Supplementary Table S5). The expression of genes involved in cardiac metabolic pathways (e.g. pyruvate, TCA cycles) were also reduced in *BFP*^−^ ACMs. However, genes for several extracellular signaling pathways such as focal adhesion assembly, extracellular matrix receptor interaction, Rap1/Integrin, and survival/proliferation-related PI3K-Akt pathways were up-regulated (Supplementary Fig. 17f–i; Supplementary Table S5). These DEGs together converged on cell survival, dedifferentiation, and proliferation pathways^29,30^. Therefore, the snRNA-seq results indicate that *BFP*^−^ ACM populations were molecularly dedifferentiated compared to the *BFP*^+^ counterparts.

As cycling and proliferative CMs were preferentially found in BFP^−^ population (Figs. 5 and 6), we further examined transcriptomic profiles of *Mki67*^+^ ACM nuclei in our snRNA-seq datasets. Among the 1939 *GFP*^+^*BFP*^−^ CM nuclei from post-infarct hearts, there were 165 (8.5%) *Mki67*-expressing nuclei, which had 681 DEGs when compared to Ki67^−^ ACM nuclei (Supplementary Fig. S18). As expected, cell cycle genes such as *Anln* and the less-studied *Knl1* and *Kif* families were significantly up-regulated in *MKi67*^+^ nuclei of dedifferentiated (*BFP*^−^) CMs (Fig. 8c). Pathway enrichment and gene set enrichment analyses revealed that most of the enriched pathways and processes were related to cell cycle activities, particularly DNA replication, mitosis, cytokinesis, and cell division (Fig. 8d; Supplementary Tables S6 and S7). Furthermore, expression of dedifferentiation genes such as *S100a6* and *Tmsb10* was higher in *BFP*^−^ (compared to *BFP*^+^) ACM nuclei, which were also significantly increased in cycling, *Mki67*^+^ ACM nuclei as compared to the *Mki67*^−^ ones (1.47 and 1.46 fold, respectively; FDR *p* < 0.05). Upstream transcription regulator analysis for the DEGs indicates that S100a6 and Foxm1 are regulators of the transcriptional reprogramming in dedifferentiated and cycling ACMs (Supplementary Fig. S19).

## Discussion

The reported discrepancies in new myocyte formation in injured hearts likely stems from multiple factors. First, the minuscule rate of new CM formation makes it challenging to measure accurately, and a small variance in quantification can result in markedly different results. Our data also confirmed that endogenous ACM cycling and renewal (e.g. Ki67^+^, Anln^+^, or BrdU^+^) is at a low rate. Second, significantly higher cycling rates of non-myocytes in the adult heart can lead to inaccurately assigning events to ACM, particularly when not using a genetic tracking model or using one with the incomplete labeling of pre-existing CMs. For instance, while MIMS is arguably the most accurate methodology currently available, it relies on the extrapolation of cell identity information derived from adjacent tissue sections^8^. An additional caveat is that conventional analyses dealing with limited numbers of cells may not have the power to reveal the very small number of scant events, i.e. proliferation of pre-existing myocytes or differentiation from putative CPCs (if any). Previous studies using direct genetic labeling of putative rare CPCs, has failed to demonstrate a significant contribution to ACM formation but have been criticized as potential being too narrow in scope, missing a significant contribution of non-myocyte to CM formation^21–24^. In this study, we employed high-throughput and readily implementable technologies, namely flow cytometry and ImageStream, to analyze total cells or total nuclei prepared from the whole ventricle. Conventional flow cytometry is widely available in modern biomedical institutes, enabling rapid data acquisition and analysis, although careful optimization is required when dealing with highly heterogeneous cells such as those in the heart. With spectrum imaging-based flow cytometry, ImageStream, we can capture the image of cell/nucleus, and ensure the rigorous identification of true positives. Instead of focusing on specific areas such as infarct or border zone that can potentially overestimate the response to cardiac injury, we used a non-biased total ventricular cell approach to assess the overall CM cycling activity in mice. Our results using these new transgenic models are the first to provide real-time visualization of CM dedifferentiation and cell cycling. Our data suggest that increased endogenous CM renewal in post-infarct hearts arise from the dedifferentiation and proliferation of pre-existing CMs and not by cardiac differentiation of putative adult CPCs.

The Cre/LoxP system allows for genetic labeling of cardiac and non-myocyte lineages by the use of conditional gene recombination in a temporal and cell type-specific manner. Many groups, including our own, previously utilized bi-transgenic αMHC-MCM;Z/EG mouse model to track the fate of CMs that switch on GFP expression after tamoxifen treatment^8,28–30^. However, this transgenic model had an incomplete GFP labeling rate (~78%) in pre-existing CMs. Incomplete genetic labeling of cell population could lead to the assignment of events to other cell types; such errors will affect the accuracy of associated analyses. For a more precise and accurate analysis of cellular activities that occur at such low rates, we developed a more efficient model for CM genetic cell fate mapping. Our data in bi-transgenic αMHC-MCM;RFP^fl^/GFP mice demonstrated highly efficient Cre/LoxP gene recombination that led to the switching of RFP expression to GFP expression in ~99% pre-existing ACMs when animals were fed with tamoxifen chow, a 27% increase from the previous model. Furthermore, GFP labeling of small, presumably non-myocyte cells in the heart was minimal (<0.03%) and showed no significant difference between sham and post-MI hearts (data not shown), confirming the tight control of gene recombination. Thus, we believe the results from this transgenic model more accurately reflect the cell sources of new CM formation in response to pathophysiological stresses such as MI.

We took advantage of the bright BFP variant^38^, and engineered a transgenic mouse with a BFP reporter specific to CM nuclei. In contrast to the previous αMHC-nLAC model expressing β-galactosidase specifically in CM nuclei that requires X-gal staining for visualization^39^, the BFP reporter in our model can be detected with or without immunostaining. This design allows greater flexibility when used in conjunction with other reporter models to detect CM events using standard high-throughput assays such as flow cytometry or high content imaging analysis^40,41^. As cell cycle progression does not affect the H2B-GFP reporter and the H2B/reporter fusion protein exchanges at a fast rate^42,43^, we postulated that a reduction in BFP signal reflects lower αMHC (*Myh6*) promoter activity in dedifferentiated CMs (Fig. 4d)^29^. Combining both mouse models into a multi-reporter tri-transgenic line allowed us for the first time to characterize cycling and molecular changes associated with dedifferentiation in vivo (Fig. 3a). The results from both bi-transgenic and tri-transgenic model suggest that pre-existing CM are the predominate resource of CM renewal, and are consistent with reports from genetic cell fate tracking of CPCs^21^. More importantly, we found that GFP^+^BFP^low^ cells morphologically and molecularly recapitulated the phenotype seen in dedifferentiated CMs. While future studies will focus on the mechanisms to control dedifferentiation, it is interesting to note that many genes up-regulated in dedifferentiated CMs are transcription factors, for example, Fosl1, Myc, Sox4, and Tmsb10. The Sox family of transcription factors may play a role in the dedifferentiation of multiple cell types and contribute to the reacquisition of primitive cell phenotypes and an enhanced cell cycle activity^30,44^. Therefore, we believe that dedifferentiation (similar to differentiation) and cell cycle activity is a molecularly regulated process in CMs.

Taking the advantages of single-cell transcriptomic analysis, we performed massive parallel cardiac snRNA-sequencing analysis that circumvented the issues associated with the significant heterogeneity of adult heart cells and the challenges in sorting a limited amount of dedifferentiated and cycling ACMs. We were able to identify specific cell populations in the heart using distinct transcriptomic clusters, transgenic reporters for ACM lineage and dedifferentiation, as well as cell cycle markers. This data also reveals typical pathological responses in post-infarct myocardium, including activation of fibrotic and inflammatory remodeling that was reflected by a panel of enriched signaling pathways and gene sets. The results demonstrated that the dedifferentiation and cell cycle progression of pre-existing CMs was augmented in post-infarct hearts, consistent with flow cytometry and ImageStream analyses. In the BFP^−^ myocyte population there was expected down-regulations of genes controlling of ACM phenotype and function; but we also discovered a number of new signaling networks that may be potentially specific to ACM dedifferentiation and cell cycle reactivation. For example, the activation of *Focal adhesion*, *Integrin/ECM receptor interaction*, *Rap1 signaling*, and *actin cytoskeleton regulation*, was seen in *BFP*^−^ ACMs (Supplementary Fig. S17, and Supplementary Tables S5, S6). These pathways play important roles in the dedifferentiation and proliferation of chondrocytes^45^, and the migration and proliferation of vascular smooth muscle cells^46,47^. We also discovered that a subset of up-regulated genes in the *BFP*^−^ and *MKi67*^+^ ACMs, such as *Knl1*, *Kif11*, and *Cdk14*, are known to promote cell cycle and proliferation in other cells. While single-cell (or nucleus) RNA-seq analysis can be limited by the sequencing depth for each cell (nucleus), we found that at least some of the DEGs identified with in vivo single-nucleus RNA-seq analyses overlap with those identified from in vitro CM dedifferentiation including *S100a6* and *Tmsb10*. Hence, targeting the related S100a6/Foxm1 signaling pathways may promote ACM dedifferentiation and cell proliferation. One caveat with cardiac single-nucleus analysis is the assumption that the nuclei within bi-nucleated or tri-nucleated myocytes are highly similar in their gene expression. The tight clustering of myocyte nuclear populations and their distinction from non-CM populations, suggests this is a reasonable assumption.

In summary, we believe that transcriptomic reprogramming, including the inactivation of gene networks governing ACM phenotype and function, together with the activation of de novo pathways and transcription factors, ultimately lead to the dedifferentiation and cell cycle progression in pre-existing CMs, giving rise to new CM formation (Fig. 8e). Given that prolonged cardiomyocyte dedifferentiation can adversely affect cardiac function^27^, knowledge of the specific regulators of both dedifferentiation and cell cycle reactivation will be required if this process is to be exploited therapeutically to promote endogenous CM proliferation without diminishing heart function.

## Materials and methods

All animals were maintained and experiments were performed in accordance with the guidelines outlined in the Public Health Service Policy on the Humane Care and Use of Laboratory Animals. Animal studies were performed under the protocol approved by the Institutional Animal Care and Use Committee at the University of Washington.

All transcriptomic data have been made publicly available at Gene Expression Omnibus under SuperSeries GSE129175, and at the ArrayExpress portal with accession number E-MTAB-3981, or from the corresponding author upon request.

The detailed “Materials and methods” section is available in the online Supplementary Information.

## Supplementary information

Supplementary Information

Supplementary Table S1

Supplementary Table S2

Supplementary Table S3

Supplementary Table S4

Supplementary Table S5

Supplementary Table S6

Supplementary Table S7

Supplementary Table S8

## Supplementary information

**Supplementary Information** accompanies the paper at (10.1038/s41421-019-0095-9).
