# Supplementary material for: Single-cell imaging and transcriptomic analyses of endogenous cardiomyocyte dedifferentiation and cycling
Source: Cell Discov. 2019 Jun 4;5:30. doi: 10.1038/s41421-019-0095-9 (PMC6547664; doi:10.1038/s41421-019-0095-9)
Supplement: Supplementary file 1 — Supplementary Information [file 41421_2019_95_MOESM1_ESM.pdf]

SUPPLEMENTARY INFORMATION

Single-Cell Imaging and Transcriptomic Analyses of Endogenous Cardiomyocyte Dedifferentiation and Cycling

Yiqiang Zhang[1,2,3*], Nuria Gago-Lopez[1,2,3], Ning Li[1,2,3,4], Zhenhe Zhang[1,2,3], Naima Alver[1,2,3], Yonggang Liu[1,2,3], Amy M. Martinson[2,3,4], Avin Mehri[1,2,3],William Robb MacLellan[1,2,3,5*]

[1]Cardiology Division, Department of Medicine, [2]Center for Cardiovascular Biology, [3]Institute for Stem Cell and Regenerative Medicine, University of Washington, Seattle, WA, USA; [4]State Key Laboratory for Biology of Plant Diseases and Insect Pests, Institute of Plant Protection, Chinese Academy of Agricultural Sciences, Beijing, China; [5]Department of Pathology, and [5]Department of Bioengineering, University of Washington, Seattle, WA, USA

*Correspondence should be addressed to:

Yiqiang Zhang, Division of Cardiology, Department of Medicine, University of Washington, 850 Republican St, Seattle, WA 98109, USA. Tel: (206) 897-1566. Fax: (206) 897-1540. E-Mail yzhang@cardiology.washington.edu, kenzhangyq@hotmail.com; or to William Robb MacLellan WRMacLellan@cardiology.washington.edu.

Supplementary Information, including:

A. **Supplementary Materials and Methods**
B. **Supplementary Figures**
C. **Supplementary Tables**

A. SUPPLEMENTARY MATERIALS AND METHODS

Bi-transgenic αMHC-MCM;RFP^{fl}/GFP mouse for cardiomyocyte lineage tracking

Bi-transgenic αMHC-MCM;RFP^{fl}/GFP mice were created by breeding αMHC-MerCreMer mouse (Jackson Laboratory; Bar Harbor, ME; Stock # 005657) with dual reporter mT/mG (here named as RFP^{fl}/GFP) mouse (Jackson Laboratory; Bar Harbor, ME; Stock # 007676)[1-4]. PCR genotyping sequences for detecting αMHC-MerCreMer transgene were: forward primer (5') ATA CCG GAG ATC ATG CAA GC (3') and reverse primer (5') AGG TGG ACC TGA TCA TGG AG (3'), yielding a 440 bp amplicon for transgene-positive littermates. Primers for RFP^{fl}/GFP detection included: wildtype forward primer (5') CTC TGC TGC CTC CTG GCT TCT (3'), wildtype reverse primer (5') CGA GGC GGA TCA CAA GCA ATA (3'), and mutant reverse primer (5') TCA ATG GGC GGG GGT CGT T (3'), with a single 250 bp amplicon for mutant (homozygous), or a single 330 bp amplicon for wildtype, or double-band (250bp and 330 bp) for heterozygous littermates. To induce cardiomyocyte-specific gene recombination, bi-transgenic mice at the age of 2-3 months were treated for 2 weeks with rodent chow containing 0.05% tamoxifen citrate per kilogram of Teklad diet (Envigo; Indianapolis, IN). Animals were then fed with standard chow without tamoxifen for at least 2 weeks before subsequently use in experiments[3, 4].

Generation of cardiac nucleus-specific reporter mice

The *BFP* gene with 6x polyhistidine tag from pB18cmBFPAzurite (a gift from Patrick Daugherty (Addgene; Cambridge, MA; Plasmid # 14034)) was subcloned into pBOS-H2B-GFP

by replacing the *GFP* gene to generate pBOS-EF1α-H2BBFP6His plasmid[5]. Subsequently, its *EF1α* promoter was replaced by full-length mouse α-myosin heavy chain (αMHC; encoded by *Mhy6*) promoter, resulting in the final pBOS-Mhy6-H2BBFP6His construct[5-7]. After the confirmation of gene construct function by transfecting pBOS-Myh6-H2BBFP6His plasmid into embryonic (E15.5) mouse myocytes in tissue culture, the *Myh6-H2BBFP6His* fragment containing polyA sequence was excised for pronuclear microinjection into fertilized B6C3 (C57BL/6 x C3H) mouse oocytes. Surviving fertilized eggs were then surgically transferred to the oviducts of pseudo-pregnant mice. Three founder transgenic *Tg(Myh6-H2BBFP)* mice (named as BFP mice) were generated. Two lines expressed BFP signal in a small percentage of CMs. However, the third transgenic mouse line CH4028 (*Tg(Myh6-H2BBFP6xHis)4028Zhy*) demonstrated robust BFP signal in all ACMs. This line was subsequently backcrossed to a C57/BL6 background to establish the stable transgenic line. BFP mice used in this study included mixed gene background as well as isogenic C57/BL6 background. To identify pups carrying the *BFP* transgene, PCR genotyping was performed with primers including: forward primer (5') GAC AGA GAA GCA GGC ACT TTA C (3'), and reverse primer (5') GAC AGA GAA GCA GGC ACT TTA C (3'), yielding a 741 bp amplicon for *BFP* transgene-positive mice. PCR primers for the internal control gene *ApoB* were: forward primer (5') CTA GGC CAC AGA ATT GAA AGA TCT (3') and reverse primer (5') GTA GGT GGA AAT TCT AGC ATC C (3'), yielding a 324 bp amplicon.

To verify the specificity of transgene expression, we bred BFP mice with RFP^{fl}/GFP mice, and examined the native BFP and RFP reporter signals. Both male and female animals were used in the experiments.

Heart weight/body weight ratio

To assess the heart weight-to-body weight ratio, aorta, fat, and other connective tissues were removed from the heart of heparinized animals, and the excess liquid was removed by blotting the heart on absorbent tissue.

Total heart cell isolation

Dissociation of total ventricular cells was performed according to an established enzymatic digestion protocol as previously described[3, 4]. Heparinized animals were anesthetized and euthanized by an overdose of isoflurane. Hearts were rapidly excised and cleansed to remove blood in ice-cold Ca^{2+}-free Tyrode's solution before being cannulated and then mounted to a Langendorff apparatus conjugated to a pressure-monitoring device. The heart was perfused retrogradely with oxygenated Ca^{2+}-free Tyrode's solution for 1-2 min to wash out blood, followed by perfusion with Ca^{2+}-free Tyrode's solution containing 0.13 Wünsch unit/mL of collagenase made from Liberase TH (Roche Molecular Biochemicals, Indianapolis, IN), for ~8 min at 37°C. The heart was subsequently perfused with normal Ca^{2+}-free Tyrode's solution for 2 min to remove the enzyme. The atria and aorta were then removed; and the ventricle was cut and minced to dissociate the heart cells. Isolated total cells in ~10 ml Tyrode's solution containing 2% fetal bovine serum (FBS) were centrifuged at 65xg for 2-3 min at room temperature to collect all CMs settled as a cell pellet. The supernatant was subsequently centrifuged at 300xg for ~5 min to collect non-myocytes. CMs and non-myocytes from each heart were resuspended and combined in Tyrode's solution, and alternatively in DPBS supplemented with 1 mmol/L $MgCl_2$. Normal Tyrode's solution contained (mmol/L): NaCl 105, KCl 5.4, KH_2PO4 0.6, NaH_2PO4 0.6, $NaHCO_3$ 6.0, $KHCO_3$ 5.0, $CaCl_2$ 1.0, $MgCl_2$ 1.0, HEPES 10.0, glucose 5.0, taurine 20.0, with pH at 7.35 (adjusted with NaOH). (−)-

Blebbistatin (25.0 μmol/L) (Toronto Research Chemicals; Toronto, ON, Canada) was added to the solutions to prevent cardiomyocytes from contraction during cell isolation steps. Chemicals were purchased from Sigma except for those specified. All tubes were pre-coated with 0.5% BSA or 2% FBS in PBS to maximize the recovery of cells.

Isolation of nuclei from the ventricles

Total heart cell nuclei were isolated from ventricles of BFP mice or wildtype littermates. The hearts were excised from heparinized animals; the blood was cleansed with ice-cold Tyrode's solution; and then the atria and connective tissues were removed. Immersed in 0.8-1.0 ml cold Tissue Lysis Buffer, ventricles were cut into fine pieces and collected into a 2-ml microtube. By using a PowerMax™ homogenizer (Model # AHS 200; VWR; Radnor, PA), tissues were homogenized on the ice at high speed for 2-3 seconds, and then rested for 5 seconds, followed by two repeats of homogenization/rest steps. The homogenate was transferred to a Type A Dounce homogenizer and homogenized gently for ~200 times on ice or until nuclei were released completely. The homogenate was further diluted into ~10 ml Tissue Lysis Buffer and then filtered through a 100 μm nylon mesh, followed by centrifugation at 700xg for 10 min at 4°C. The pellet was then resuspended in 10 ml Tissue Lysis Buffer, filtered through a 40 μm mesh, and then centrifuged at 700xg for 10 min at 4°C. A last step of filtration with a 20 μm mesh and then centrifugation at 700xg for 10 min yielded a crude nuclei pellet that was resuspended in 2 ml of Tissue Lysis Buffer. Enriched nuclear preparations were obtained by loading the resuspended crude nuclei onto the top of a 3-ml Sucrose Cushion solution in a 5-ml microtube, followed by centrifugation at 700xg for 10 min at 4°C. Nuclei were washed and resuspended in Nuclei Storage

Buffer as an unfixed preparation, or followed by fixation steps. To fix nuclei, each ventricular preparation was resuspended in 20 ml cold DPBS in a pre-coated 50-ml centrifuge tube containing 1mM $MgCl_2$ and 0.5% NP-40, then mixed with same volume of cold DPBS containing 1.0 mmol/L $MgCl_2$, 0.5% NP-40 and 2% paraformaldehyde, for 30 min at room temperature, with the container being rotated and agitated to prevent aggregation. The preparations were protected from light during the fixation steps. After wash, fixed nuclei were cryopreserved in 90% FBS supplemented with 10% DMSO at -80°C. Tissue Lysis Buffer contained (mmol/L): D-sucrose 320, $CaCl_2$ 2, $MgCl_2$ 2, Tris-HCl 10, pH 7.4 (adjusted with Tris base). Sucrose Cushion Solution contained: D-sucrose 1.0 M, $CaCl_2$ 2 mM, $MgCl_2$ 2 mM, Tris-HCl 10 mM, pH 7.4 (adjusted with Tris base). Nuclei Storage Buffer contained (mmol/L): D-sucrose 430, KCl 70, $MgCl_2$ 2, Tris-HCl 10, pH 7.4 (adjusted with Tris base). Except for fixation steps, all solutions were supplemented with additives including: EDTA-free protease inhibitors (Thermo Fisher Scientific, Inc.), NaF 1.0 mmol/L, Na_3VO_4 1.0 mmol/L, Phenylmethylsulfonyl fluoride (PMSF) 1.0 mmol/L, 2-Mercaptoethanol 0.1%, Spermine 2.0 mmol/L, and NP-40 0.5% (or 0.1% when used in Nuclei Storage Buffer or buffers used for immunostaining).

Isolation of cells and nuclei from small intestines

To isolate total small intestine cells as control cells, small intestine in ~25-30 mm was cleansed gently with normal Tyrode's solution, then cut and minced into fine pieces, followed by 4 repeats of 2-3 min digestions at 37°C with ~5 ml of Liberase TH (0.13 Wünsch unit/mL of collagenase; Roche Molecular Biochemicals; Indianapolis, IN). Dissociated cells were combined in ice-cold Tyrode's solution containing 2% FBS and then filter through cell strainers with a pore

size of 70 μm, 40 μm then 20 μm. Small intestine cells were then fixed with 4% paraformaldehyde in DPBS for 10 min at room temperature.

To isolate small intestine nuclei, cleansed 25-30 mm small intestine tissue was minced into fine pieces in Tissue Lysis Buffer containing all additives as described for the isolation of heart nuclei. The tissue was homogenized on ice at high speed for 2-3 seconds with a PowerMax™ homogenizer (Model # AHS 200; VWR; Radnor, PA), followed by ~60 times of dounces in a glass homogenization to release nuclei from the cells. Subsequent steps were followed similarly as those for total heart nuclei preparation.

Myocardial infarction model

Acute myocardial infarction (MI) was made in adult mice by permanent ligation of the left anterior descending coronary artery (LAD) as described previously[4]. To label cells with DNA synthesis activity (e.g. S phase in the cell cycle), BrdU (5-Bromo-2'-deoxyuridine) or EdU (5-ethynyl-2'-deoxyuridine) (0.4 mg/ml) in drinking water was given to animals ad. lib, continuously starting at ~15 hr post-surgery.

Immunocytochemistry and flow cytometry assays

For total heart cell preparations, ventricular cardiomyocytes and non-myocytes from each heart were resuspended and combined in 40 ml of fixation solution containing 4% paraformaldehyde in DPBS supplemented with 1.0 mmol/L $MgCl_2$ and 0.1% NP-40. Alternatively, combined cells were resuspended in 1.0 ml DPBS supplemented with 1.0 mmol/L $MgCl_2$, then fixed in 20 ml methanol containing 1.0 mmol/L $MgCl_2$, at -20°C for 10 min. The cells were

protected from light during the fixation steps. Fixed cells were aliquoted and cryopreserved at -80°C in 90% FBS supplemented with 10% DMSO. The native fluorescent reporter signals were retained in cells after these procedures. The following primary antibodies were used: anti-αMHC from Santa Cruz Biotechnology, Inc (Dallas, TX; #sc-168676), anti-α-sarcomeric actinin (α-SA) from Sigma-Aldrich (St. Louis, MO; #A2172), anti-tropomyosin I from Abcam (Cambridge, MA; #ab55915), anti-cardiac troponin T (cTnT) from Santa Cruz Biotechnology, Inc (#sc-8121), anti-cardiac troponin I (cTnI) from Abcam (#ab47003), anti-GFP from Aves Lab (Tigard, OR; #GFP-1020) or Rockland Immunochemicals Inc (Pottstown, PA; #RL600-141-215), anti-6X His tag from Abcam (#ab9136) or Rockland Immunochemicals Inc (#RL200-346-382), anti-RFP from Biotium, Inc. (Fremont, CA; #20477), and anti-pericentriolar material 1 (Pcm1) from Abcam (#ab72443). Secondary antibodies conjugated with Alexa Fluor were used to detected protein expression as described previously[3, 8].

To detect BrdU incorporation, a modified protocol from a BD Bioscience kit was used [9]. Briefly, cells of each cryopreserved aliquot (about 1/10 of total ventricular cell preparation) were washed with DPBS, resuspended in a siliconized microtube and then re-fixed by 0.5 ml 4% paraformaldehyde in DPBS containing 0.1% Triton X-100 and 1.0 mmol/L $MgCl_2$, for 15 min at room temperature; protected from light. During cell fixation, the tubes were rotated to prevent cells from aggregation. After centrifugation at 400xg for 3 min in a swing bucket, the cells were subsequently washed with 1ml DPBS containing 5% FBS, for three times. The cells were then incubated at 37°C for 45 min with 0.2 ml DNase I in DPBS (300 μg/ml; Sigma-Aldrich; #D-4513), with microtubes rotated continuously. Subsequently, the cells were washed with 0.3 ml 1x BD Perm/Wash Buffer for 2 times, followed by immunostaining with PE-, APC-, Alexa Fluor 647, or APC-Cy7-conjugated anti-BrdU antibody (Catalog # 51-35405X-2, #552598, #560209 from BD

Biosciences, and AC12-0250-05 from Abcore Inc. (Ramona, CA), respectively). Proper isotype controls were used to confirm the specificity of antibodies. Nuclei in cells from BFP or tri-transgenic mice were stained with Draq5 (Thermo Fisher Scientific) or Draq7 (Abcam), while nuclei in cells from bi-transgenic αMHC-MCM;RFP^{fl}/GFP mice were stained with DAPI, Draq5, or Draq7.

To detect EdU incorporation, Click-iT EdU Alexa Fluor 555 HCS Assay (Thermo Fisher Scientific Inc.) with the modified protocol was used. For each sample, about one-tenth of total ventricular cells or nucleus preparation was used for one Click-iT EdU reaction (in ~0.4 ml of the reaction mixture for cell preparation, and 0.2 ml for nucleus preparation). The native BFP fluorescent signal was quenched in this EdU detection assay due to the presence of Cu^{2+} in the reaction mix, but it was recovered by immunostaining against BFP or 6xHis tag. Daily EdU label rate (E) was calculated using the formula $E = \left(\sqrt[Accum.\,Days]{1 + accum.\ EdU\ Rate} - 1\right) \text{x } 100\%$, where *Accum.Days* is the total days of cumulative labeling, and *accum.Edu Rate* is the cumulative EdU labeling rate.

Other antibodies for cell cycle included: Alexa Fluor 647-conjugated mouse anti-Ki67 antibody (BD Biosciences; 558615), APC-Cy7-conjugated goat Anti-Anillin antibody (Abcore Inc; AC21-0953-05).

Digital flow cytometry was performed on BD Canto RUO II or LSR II flow cytometer. Control cells or nuclei of non-color and single-color, and isotype antibody controls, were used to set up proper instrument parameters. Prior to flow cytometry analysis, cell and nucleus preparations were filtered through a 100 µm and a 20 µm mesh, respectively. A-, H-, and W-indices were collected for forward scatter (FSC), side scatter (SSC), and all fluorescent channels.

The channel of nuclear dye staining was mostly set in linear scale and other fluorescent channels were set in log scale. For total heart cell analyses, FSC scaling factor was set to 0.1 to 0.2, and both FSC and SSC were set in log scale, thereby we could better cluster the larger cells (i.e. cardiomyocytes) and the smaller cells (i.e. non-cardiomyocytes). About 50,000 cell events were collected for each sample run. In a subset of the analyses, we defined BFP reporter signal as BFP^{low} if it was negative and as BFP^{high} if it was positive when compared to control samples.

Fluorescence-activated cell sorting (FACS)

Fixed and cryopreserved heart cells were retrieved from -80˚C storage and washed three times with DPBS containing 1.0 mM $MgCl_2$ and 2% FBS. FACS Aria II (BD Biosciences) cell sorter was used to sort GFP^+BFP^{high} and GFP^+BFP^{low} (GFP^+BFP^-) myocytes. Nuclear dye Draq5 was used to identify cells. Control heart cells that were non-florescent, or BFP^+, GFP^+ or RFP^+, were used to set up proper instrument parameters.

ImageStream analysis (Imaging flow cytometry)

After immunostaining, cells were resuspended in DPBS containing 1% FBS, filtered through a 100 µm nylon mesh, then the solution was reduced to ~150 µl for each cell preparation that was initially ~1/10 of cells or nuclei of the whole ventricle. Aliquots in 20-30 µl were loaded for analysis with an ImageStream X Mark II imaging flow cytometer system (Millipore/Amnis) with a 10-µm core at 66 mm/sec, and with 7% SpeedBeads that were used for the calibration of the flow and focus of the ImageStream system. The accompanied INSPIRE software (version 200; Millipore/Amnis) was used for data acquisition. Between each sample load (20-30 µl), following

a 2-min run of bleach, the system was purged to eliminate contaminations from the previous sample. Fluorochromes were excited with 405, 488, 642 nm lasers and light emitted by fluorescently-labeled cells were collected through a 20x objective in the "High Sensitive" setting. SSC was set in Channel Ch06 that was lighted by a 785 nm laser on the particles (cells). Alternatively, Ch06 was assigned to APC-Cy7 signal, with a 642 nm laser for excitation, and a bandwidth of 762/35 nm for emission collection. To prevent cardiomyocytes from settling in sample lines of the system, the sample loading lines were tapped gently every ~3 minutes. Data from single-color samples were used to generate a compensation matrix using IDEAS software (version 6.2; Millipore/Amnis). Data from multiple runs of a sample were merged for offline analysis. Multiple features such as Threshold, Intensity, Range, Watershed, were combined to generate optimal masks for each channel. To analyze cardiomyocyte dedifferentiation state, we classified BFP signal based on the Median Pixel values on Ch01, with a value <1.5x threshold defined as "no/low" BFP signal (BFP^{low}), and a value ≥1.5x threshold as "high" BFP signal. Reported data were generated from visually verified cell events after proper gating.

Fluorescent immunohistochemistry and high content imaging analysis (HCIA)

Ventricular sections in 14 μm were prepared by using a Cryostat (Leica CM1850). Fluorescent immunohistochemistry was performed to detect Ki67 expression and BrdU incorporation in cardiomyocytes. Tissues were fixed with 4% paraformaldehyde in DPBS containing 1.0 mM $MgCl_2$ for 30 minutes at room temperature, permeabilized with 0.1% Triton X-100 for 15 minutes, then blocked with 5% normal donkey serum (Jackson ImmunoResearch Laboratories, Inc., West Grove, PA). For BrdU immunostaining, DNase I treatment was performed similarly to isolated cells as described in previous sections. Alexa Fluor 647-conjugated mouse

anti-Ki67 and anti-BrdU antibody (BD Biosciences; #558615, #560209, respectively) were used to detect Ki67 expression and BrdU incorporation.

Whole tissue sections were imaged using Nikon TiE inverted widefield fluorescence microscope or spinning disc confocal microscope with the "Scan Large Image" function of accompanying Element software (version 4; Nikon). To analyze nuclear profile, acquired images were analyzed by using either "Object Count" function in Element software or "Analyze Particles" function in ImageJ software (version 1.50i; NIH, USA) for the images of individual fluorescent channels. Optimal thresholds were set for nuclear staining and molecules of interest (e.g., BFP, or Pcm1) prior to object counting and signal intensity normalization.

Quantification of BFP signal in post-infarct tri-transgenic hearts

To quantify the BFP signal in CMs within infarct scar, border, or remote zones, we converted single-color fluorescent images of BFP, GFP, RFP reporters and nuclear dye (Draq5) into binary images with ImageJ software. Border zone was defined as within 150 μm from the edge of infarct scar. The masks of nuclear staining for GFP^+ CMs were applied to BFP channel of the corresponding fields. The total BFP signal within the nuclear mask of GFP CMs was normalized to the total DNA dye signal, by which we calculated the normalized BFP signal strength and the percentage of BFP^- CMs.

Bioinformatic analysis of ACM in vitro dedifferentiation-specific genes based on the comparison of myocyte types

In this study, we utilized both our published Affymetrix Mouse Genome 430 2.0 Array data of *in vitro* ACM dedifferentiation[4, 10], and transcriptomic data comparing embryonic, adult, and hypertrophic cardiomyocytes (ECM, ACM and TAC, respectively) that were generated by using Illumina BeadChip arrays. Microarray data can be accessed at the Gene Expression Omnibus portal with the following accession numbers: GSE129090 for Illumina MouseRef-8 v2.0 Expression BeadChip data, and GSE49448 for Affymetrix transcriptomic data (also available in ArrayExpress under accession number E-MTAB-3981)[4, 10]. Details on data acquisition and bioinformatics analysis of Affymetrix mouse transcriptome comparing control ACMs and dedifferentiated ACMs *in vitro* can be found in references[3, 4, 10]. For Illumina Expression BeadChip data, ECM, ACM, and TAC were isolated from 2, 3, and 3 mice, respectively. Total CM RNA for each heart was isolated using a RNeasy kit (Qiagen). cDNA library was prepared according to Illumina BeadChip protocols. Standard microarray hybridization, scanning, and data acquisition protocols were followed according to manufacture manuals (Illumina). Gene feature data were extracted from array images using GenomeStudio software (version 2.0; Illumina). To avoid the issues of platform differences between the two transcriptome datasets, no inter-platform normalization were performed. Partek Genomic Suite was used to perform differential gene expression analysis as reported previously[4, 10]. The genes with $|\text{fold-change}| \geq 2$ (FDR-adjust $p<0.05$) between groups were defined as differentially expressed genes (DEGs). After DEG finding in the two datasets, Venn diagrams were used to generate DEGs specific to a certain CM type. Our bioinformatics analysis revealed that dedifferentiated cardiomyocytes have distinct transcriptome profiles comparing to other types of cardiomyocytes. Dedifferentiated cardiomyocyte-specific genes were defined as those that were up-regulated when compared to ACMs, but not overlapped

with those genes that were up-regulated in ECMs compared to ACMs (Supplementary Fig. S11; and Supplementary Table S1).

RT-qPCR analysis of dedifferentiated cardiomyocyte-specific genes

To assess gene expression in $GFP^{+}BFP^{high}$ ($GFP^{+}BFP^{+}$) and $GFP^{+}BFP^{low}$ ($GFP^{+}BFP^{-}$) cardiomyocytes, total RNA was isolated from sorted cells (~2,000-5,000 cells) using a modified protocol for fixed cells based on RNeasy FFPE kit (Qiagen). Transcriptor First Strand cDNA Synthesis Kit (Roche Life Science) with dT18 primer and random hexamer primer option for reverse transcription was used to synthesize cDNA from total RNA. SYBR® Green Real-Time PCR Master Mix was used in qPCR reactions that were performed on the 7900HT Fast Real-Time PCR System (Applied Biosystems). Raw data was collected and analyzed using SDS 2.4 software suite (Applied Biosystems). Ct values were normalized to that of *Gapdh*, and comparative $2^{-\Delta\Delta Ct}$ method was used to evaluate relative gene expression in $GFP^{+}BFP^{low}$ versus $GFP^{+}BFP^{high}$ cardiomyocytes. Relative Expression Software Tool (REST; version 2.0; Qiagen) was used to analyze the changes of gene expression. The primer sequences for genes in ACM dedifferentiation analysis are listed in Supplementary Table S8.

Massive parallel single-nucleus transcriptomic analysis

Nuclei preparation — To determine the molecular signatures and pathways enriched in dedifferentiated cardiomyocytes in post-infarct hearts, we adopted massive parallel single-nucleus RNA-seq analysis using 10x Genomics platform. One control and two 5-days post-infarct mice were used in the analysis. Fresh frozen ventricular tissues from triple-transgenic mice at the early

post-infarction time point (5-day) were stored at -80˚C and used for nuclei preparation. Ventricle sample was cut into small pieces and ground into fine powders in a motor chilled with liquid N_2. The samples were added with 2.5 ml Cell Lysis Buffer containing 10mM Tris-HCl, 10mM NaCl, 3 mM $MgCl_2$, 0.1% IGEPAL, 0.2 Unit/µl RNase inhibitor (Thermo #AM2696), 20 µl/ml Protease inhibitor (Sigma P8340), 0.1% BSA, and 2.0 mM Spermine (pH 7.4), and dounced in a B-type homogenizer until all intact nuclei were released. The homogenates were diluted with 1.5 ml lysis buffer before filtered through a 40 µm mesh. Additional 2.0 ml Cell Lysis Buffer was used to rinse the meshes and eluents were combined, followed by centrifugation at 500xg for 5 minutes at 4˚C. Crude nuclei pellets were resuspended in 1.5 ml Cell Lysis Buffer, then filtered through a 20 µm mesh. The filtrates were centrifuged at 1,000xg for 3 minutes at 4˚C. Nuclear pellets were resuspended in 1.0 ml Nuclear Loading Solution containing (mM): 250.0 Sucrose, 25.0 KCl, 5.0 $MgCl_2$, 20.0 Tris-HCl 20, and 2.0 Spermine. Subsequently, resuspended nuclei were purified by passing through a 29% Iodixanol layer with centrifugation at 1,000 xg for 10 min at 4 ˚C. Recovered nuclei were washed, then resuspended in Nuclear Buffer supplemented with RNase inhibitor and 0.1% BSA. Nuclei used for 10x Genomics single-nucleus capture were be diluted to 700-1,200 nuclei/µl in PBS supplemented with RNase Inhibitor 0.2U/µl, BSA 0.2%, and $MgCl_2$ 3.0 mM.

Single-nucleus RNA-seq library — Single-nucleus libraries were generated using the Chromium Controller and Single Cell 5' Library & Gel Bead Kit and Chip A Kit (10x Genomics) according to the manufacturer's protocol. Briefly, 17.4 µl nuclear stock was mixed with 16.4 µl water for each sample to obtain reaction mixtures that were loaded to the microfluidic Chip A. Reverse transcription to generate barcoded cDNA library for each nucleus were performed in the resultant Gel-bead-in-EMulsions (GEMs). Subsequently, single-nucleus droplets were broken and

the single-strand cDNA was isolated and cleaned with DynaBeads provided in the kit, and then amplified using the recommended thermal cycle protocol. Amplified cDNA then was fragmented, end-repaired, and A-tailed, and ligated with sample index adaptor, then PCR amplified to generate the sequencing-ready library. SPRIselect Reagent Kit for cleanup was used in between steps. Gene expression libraries were ligated with Index adaptors. Paired-end (26bp x 8bp x 98bp) sequencing was performed on HiSeq 2500 with high output mode (Illumina).

Single-nucleus RNA-seq data analysis — Cell Ranger package (10x Genomics; version 3.0) was used to process the single-nucleus RNA-seq data. Pre-mRNA transcriptome integrated with the four transgenes (*Cre* transcript within the MerCreMer fusion gene, *BFP*, *RFP*, and *GFP*) was built using *cellranger mkref* command according to 10x Genomics' recommendation. Mouse GRCm38.93 fasta and transcript annotations were downloaded from Ensembl, including a subset of "gene" types such as protein coding, lincRNA, antisense. Transgenes were added to both as new contigs in the fasta file and as genes/transcripts, and subsequently, a mouse pre-transcriptome reference was generated by writing a new annotation file. *Cellranger count* was used to obtain gene-level summaries. For the one control heart and the two post-infarct hearts, the pipeline produced 9,519, and 11,211 and 13,801 nuclei, respectively, that contained valid barcodes; and all matrices were proximal to the single-cell gene expression matrices published by 10x Genomics. After comparing the matrices of two 5-days post-infarct heart data, we pooled the results by using *cellranger aggr* function, with normalization step implemented prior to the aggregation according to the 10x Genomics recommendation. Loupe Cell Browser (10x Genomics; Version 3.0), R package Seurat (Version 3.0)[11] and Partek Flow (Partek, Inc.) were used for outlier removal, normalization, imputation, gene filtering, dimensional reduction, clustering and visualization, differential gene expression and protein network analyses. We filtered out the nuclei with extreme

amounts of UMI counts or detected genes, and set the mitochondrial count level in nuclear datasets at a limit of 2%. Data were normalized by three steps: count per million, then adding 1, followed by log 2 normalization. The gene features that had a zero value in all the cells were excluded from subsequent data analysis. Positive expression of a gene (e.g. reporter gene *GFP*, *RFP*, or *BFP*) was defined by its normalized value ≥1 (a minimal UMI=1); and negative expression, value <1. ANOVA analysis was used to compare the difference between classes of nuclei with specific transcript markers. Gene ontology and pathway enrichment analyses were performed using Partek Flow (Partek Inc.) by uploading the gene lists to the integrated online portal as previously described[4, 10, 12]. In this study, we focused on two major classes of cells in post-infarct hearts: $GFP^{+}RFP^{-}BFP^{+}$, $GFP^{+}RFP^{-}BFP^{-}$, and compared the $Mki67^{+}$ versus $Mki67^{-}$ population, so to dissect transcriptomic reprogramming of cardiomyocyte dedifferentiation and cell cycle activity in post-infarct hearts. Axis labels for 2-dimensional t-SNE plots are omitted for concise presentation in the figures.

Statistics

Statistical comparisons and graphic presentation were carried out using Prism software (version 6; GraphPad Software; La Jolla, CA). Group data were expressed as mean±S.E.M. Comparisons were performed via an unpaired (or paired), two-tailed Student's t-test, one-way ANOVA test followed by Tukey multiple-comparisons test, with significant differences defined by * $p< 0.05$, ** $p< 0.01$, and *** $p< 0.001$. For RT-qPCR, a significant difference in gene expression was defined by * $p<0.05$ with hypothesis test (REST software; Qiagen).

Study Approval

All animals were maintained and experiments were performed in accordance with the guidelines outlined in the Public Health Service Policy on the Humane Care and Use of Laboratory Animals. Animal studies were performed under the protocol approved by the Institutional Animal Care and Use Committee at the University of Washington.

Data deposition

Transcriptomic data in this study is available at Gene Expression Omnibus (GEO) under SuperSeries GSE129175. The microarray data for dedifferentiated ACMs versus normal ACMs are accessible at ArrayExpress portal with accession number E-MTAB-3981[4, 10] or at Gene Expression Omnibus (GEO) under accession numbers GSE49448. The Illumina BeadChip array data for gene expression in ECMs, ACMs and hypertrophic (TAC) myocytes is under accession number GSE129090, and snRNA-seq dataset is under accession number GSE128628, or from the corresponding author upon request.

Supplemental References

1. Muzumdar,M.D., Tasic,B., Miyamichi,K., Li,L., & Luo,L. A global double-fluorescent Cre reporter mouse. *Genesis.* **45**, 593-605 (2007).
2. Sohal,D.S. *et al.* Temporally regulated and tissue-specific gene manipulations in the adult and embryonic heart using a tamoxifen-inducible Cre protein. *Circ. Res.* **89**, 20-25 (2001).
3. Zhang,Y. *et al.* Dedifferentiation and proliferation of mammalian cardiomyocytes. *PLoS. One.* **5**, e12559 (2010).
4. Zhang,Y. *et al.* Epigenomic Reprogramming of Adult Cardiomyocyte-Derived Cardiac Progenitor Cells. *Sci. Rep.* **5**, 17686 (2015).

5. Mena,M.A., Treynor,T.P., Mayo,S.L., & Daugherty,P.S. Blue fluorescent proteins with enhanced brightness and photostability from a structurally targeted library. *Nat. Biotechnol.* **24**, 1569-1571 (2006).

6. Kita-Matsuo,H. *et al.* Lentiviral vectors and protocols for creation of stable hESC lines for fluorescent tracking and drug resistance selection of cardiomyocytes. *PLoS ONE* **4**, e5046 (2009).

7. Subramaniam,A. *et al.* Tissue-specific regulation of the alpha-myosin heavy chain gene promoter in transgenic mice. *J. Biol. Chem.* **266**, 24613-24620 (1991).

8. Gago-Lopez,N. *et al.* THY-1 Receptor Expression Differentiates Cardiosphere-Derived Cells with Divergent Cardiogenic Differentiation Potential. *Stem Cell Reports.* **2**, 576-591 (2014).

9. Malliaras,K. *et al.* Cardiomyocyte proliferation and progenitor cell recruitment underlie therapeutic regeneration after myocardial infarction in the adult mouse heart. *EMBO Mol. Med.* **5**, 191-209 (2013).

10. Chen,X. *et al.* Single-cell transcriptome and epigenomic reprogramming of cardiomyocyte-derived cardiac progenitor cells. *Sci. Data* **3**, 160079 (2016).

11. Butler,A., Hoffman,P., Smibert,P., Papalexi,E., & Satija,R. Integrating single-cell transcriptomic data across different conditions, technologies, and species. *Nat. Biotechnol.* **36**, 411-420 (2018).

12. El-Nachef,D. *et al.* Repressive histone methylation regulates cardiac myocyte cell cycle exit. *J. Mol. Cell Cardiol.* **121**, 1-12 (2018).

B. SUPPLEMENTARY FIGURES

Supplementary Fig. S1

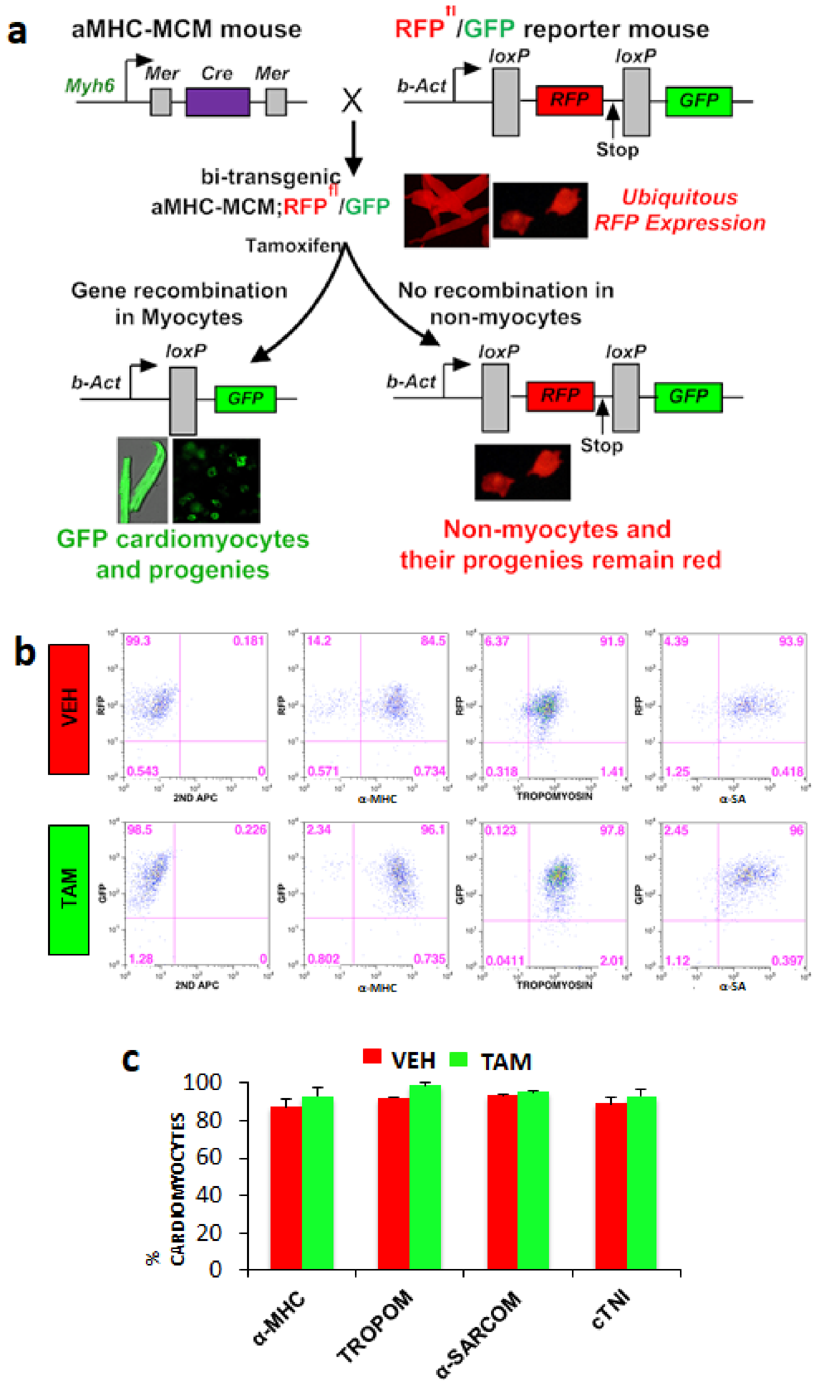

Supplementary Fig. S1. Improved Cre/LoxP system with specific and efficient cardiomyocyte genetic labeling. a, Scheme of transgene constructs and Cre/LoxP-mediated gene recombination for cardiomyocyte fate tracking. Postnatal cardiomyocytes and their progenies will constitutively express membrane-targeted GFP driven by β-actin promoter (*b-Act*) after the excision of floxed RFP gene mediated by tamoxifen-inducible Cre recombinase activity that is under the control of cardiac-specific αMHC (*Myh6*) promoter. Non-myocytes will not have Cre/LoxP gene recombination, therefore, they remain expressing RFP, and so do their progenies. **b,** Flow cytometry analysis of cardiac markers (α-Myosin heavy chain (α-MHC), Tropomysin (Tropom), and α-Sarcomeric actinin (α-SA) in myocytes expressing RFP (Vehicle-treated mice; VEH) or GFP (Tamoxifen-treated mice; TAM). **c,** Expression of cardiac markers in myocytes with RFP (VEH treated) or GFP (TAM treated) reporter. cTNI, Cardiac Troponin I. n=4 mice for each group.

Supplementary Fig. S2

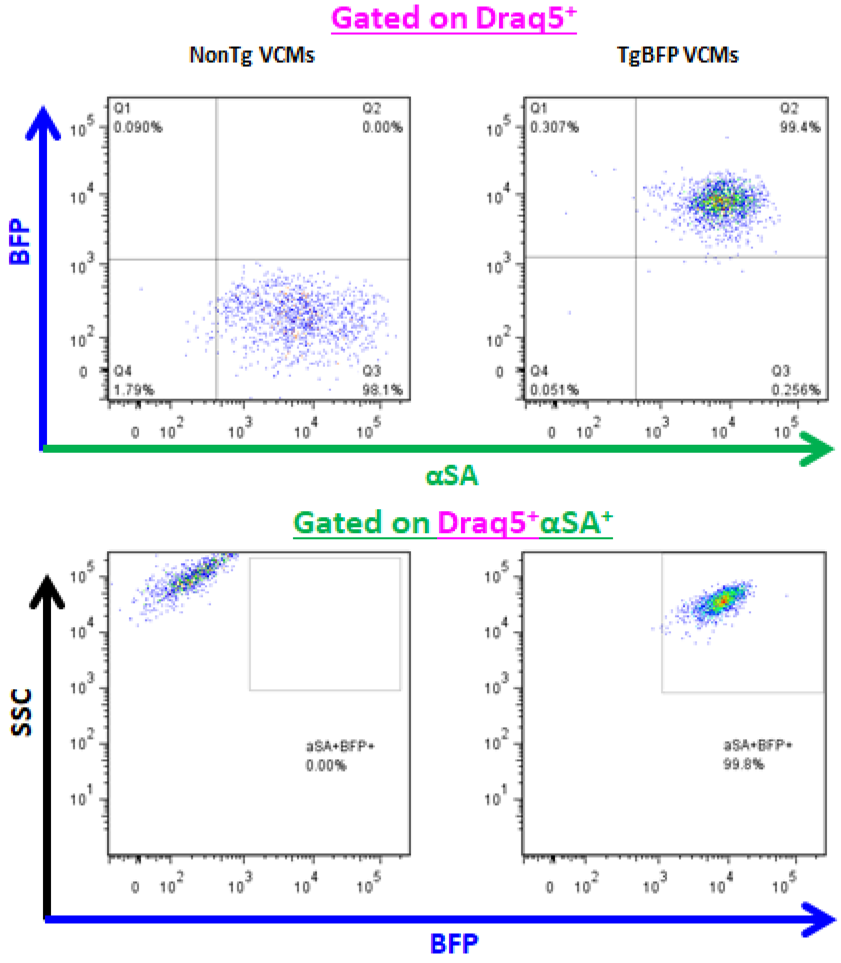

Supplementary Fig. S2. Efficient BFP expression in cardiomyocytes. Flow cytometry analysis showing the expression of native BFP signal in transgenic BFP mouse (TgBFP) ventricular cardiomyocytes (VCM) that are positive to α-sarcomeric actinin (αSA) as revealed by Alexa Fluor 488 conjugated second antibody. Total ventricular cell nuclei were stained with Draq5, and then gated on αSA to analyze the expression of BFP. BFP-positive cells accounted for 99.1±0.7% (n=3) among αSA-positive myocytes. VCMs from non-transgenic littermates (NonTg) were used to set up the gates.

Supplementary Fig. S3

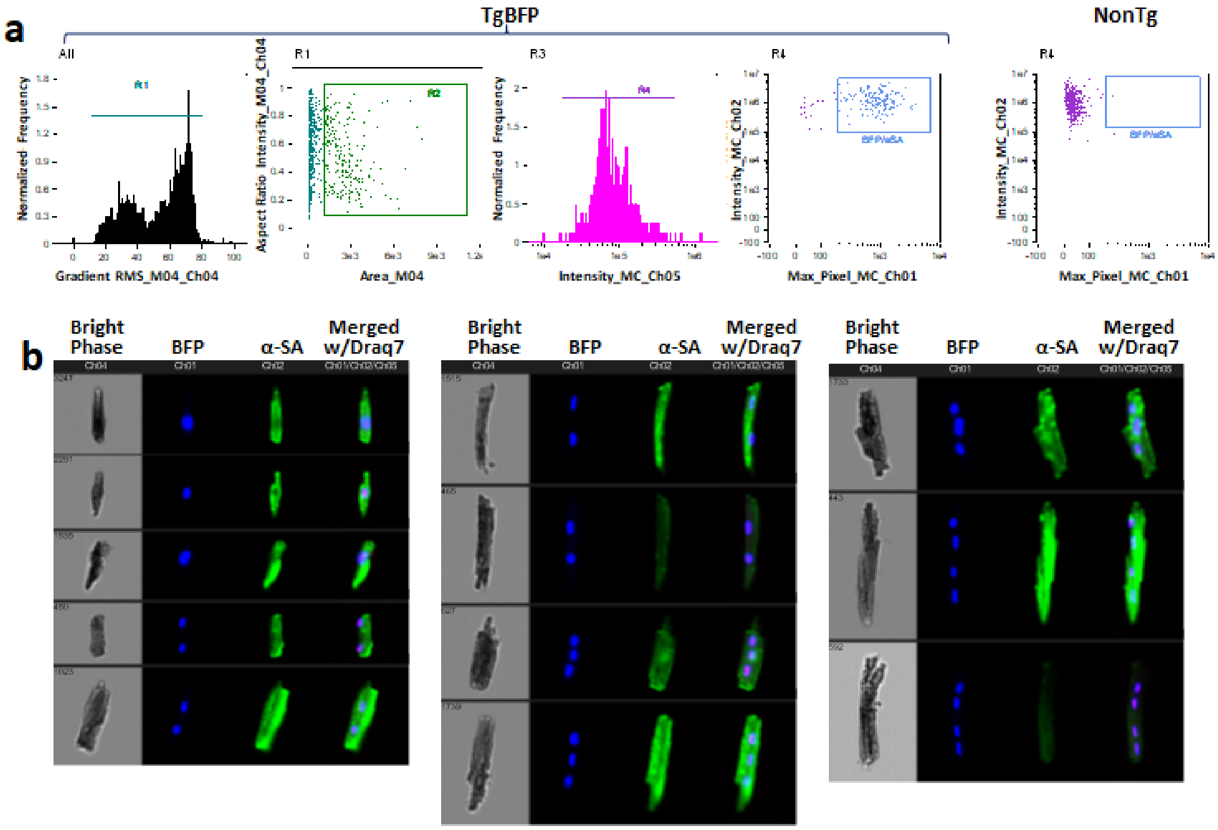

Supplementary Fig. S3. Co-expression of BFP and α-sarcomeric actinin (α-SA) in transgenic BFP mouse (TgBFP) ventricular cardiomyocytes. Analyzed using ImageStream flow cytometry, BFP signal (blue) was in Channel 01; α-SA (green) in Channel 02; nuclei were stained by Draq7 (Channel 05); and the bright field was set to Channel 04. **a**, ImageStream gating strategy. The expression of α-SA and BFP were analyzed in large cells (R2) with Draq7 staining (R4). The right panel is the dot plot for cells from a non-transgenic littermate. **b,** Example images of individually imaged TgBFP mouse cardiomyocytes with different nucleation. All cells were gated as double-positive to BFP and α-SA as shown in panel **a**.

Supplementary Fig. S4

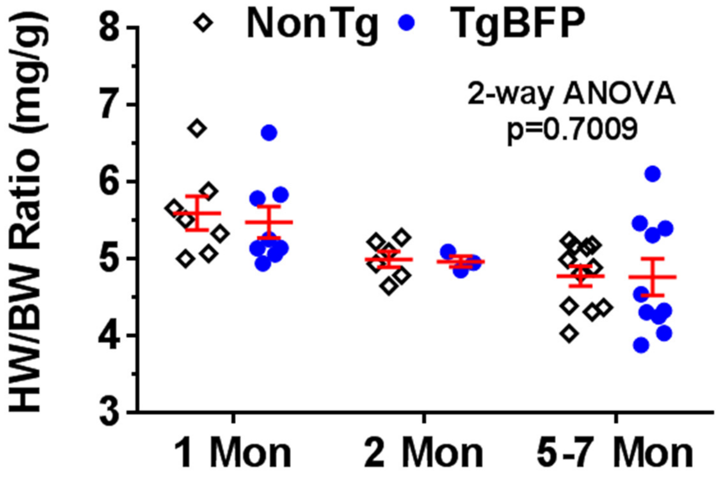

Supplementary Fig. S4. No difference of heart weight-to-body weight ratios between transgenic (TgBFP) and non-transgenic (NonTg) littermates at different ages. Littermates from mixed gene backgrounds were used. Shown are individual mouse data points.

Supplementary Fig. S5

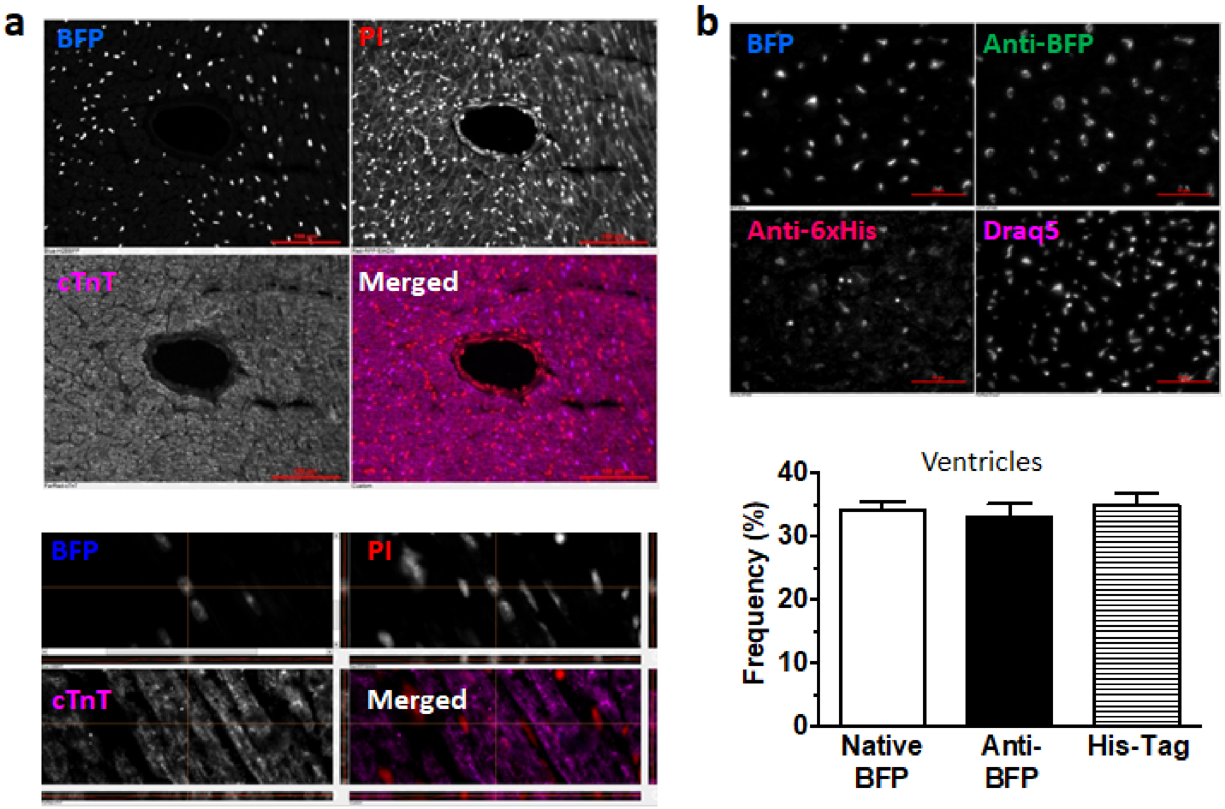

Supplementary Fig. S5. Specific transgenic expression of BFP and histidine tag in cardiomyocytes. a, *Upper panels*: High content image analysis of the expression of native BFP signal (Blue) in ventricular sections from transgenic BFP mice. Cardiomyocytes were identified by cardiac troponin T (cTnT, magenta) and nuclei were stained by propidium iodide (PI, red). *Lower panels*: Confocal images with an orthogonal view of the ventricular section showing the expression of native BFP (blue) and cTnT (magenta), with nuclei stained by Propidium iodide (PI; red). **b**, Images showing the expression of native BFP fluorescent signal (blue), immunoreactive BFP signal (anti-BFP, green) and immunoreactive 6xHis-tag signal (red) in ventricular tissue of BFP mouse. Draq5 (magenta) was used to stain the nuclei. *Bar chart*, percentage of nuclei with signals of native BFP, anti-BFP or anti-His-Tag in total ventricular nuclei. There is no significant difference between groups ($p>0.05$, ANOA test; n=3 mice).

Supplementary Fig. S6

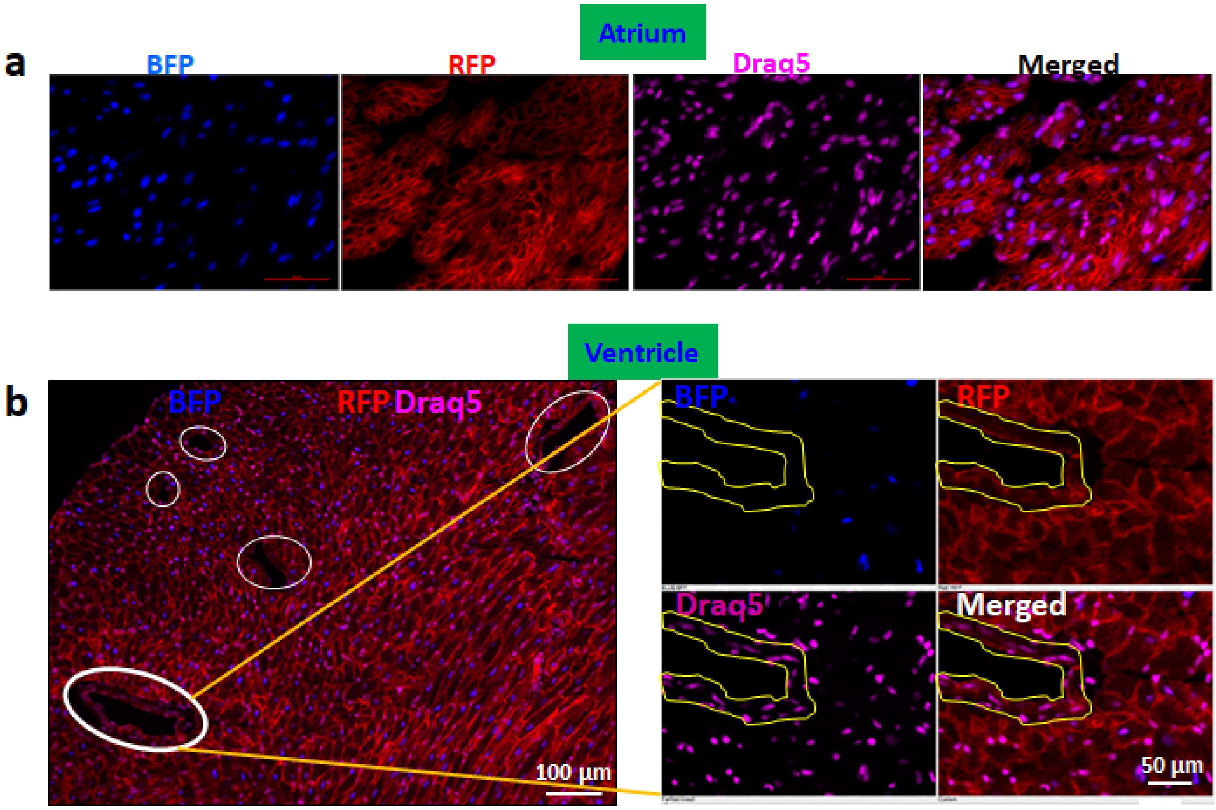

Supplementary Fig. S6. Cardiac Specific Expression of BFP in double reporter BFP;RFP^{fl}/GFP mice. Images show the expression of BFP in the atrium (**a**) and the ventricle (**b**). BFP signal is not detectable in the vessels (white circles and yellow trace marks). Both BFP and RFP are native fluorescent signals. Nuclei were stained by Draq5 (magenta). RFP signal expresses in all cells.

Supplementary Fig. S7

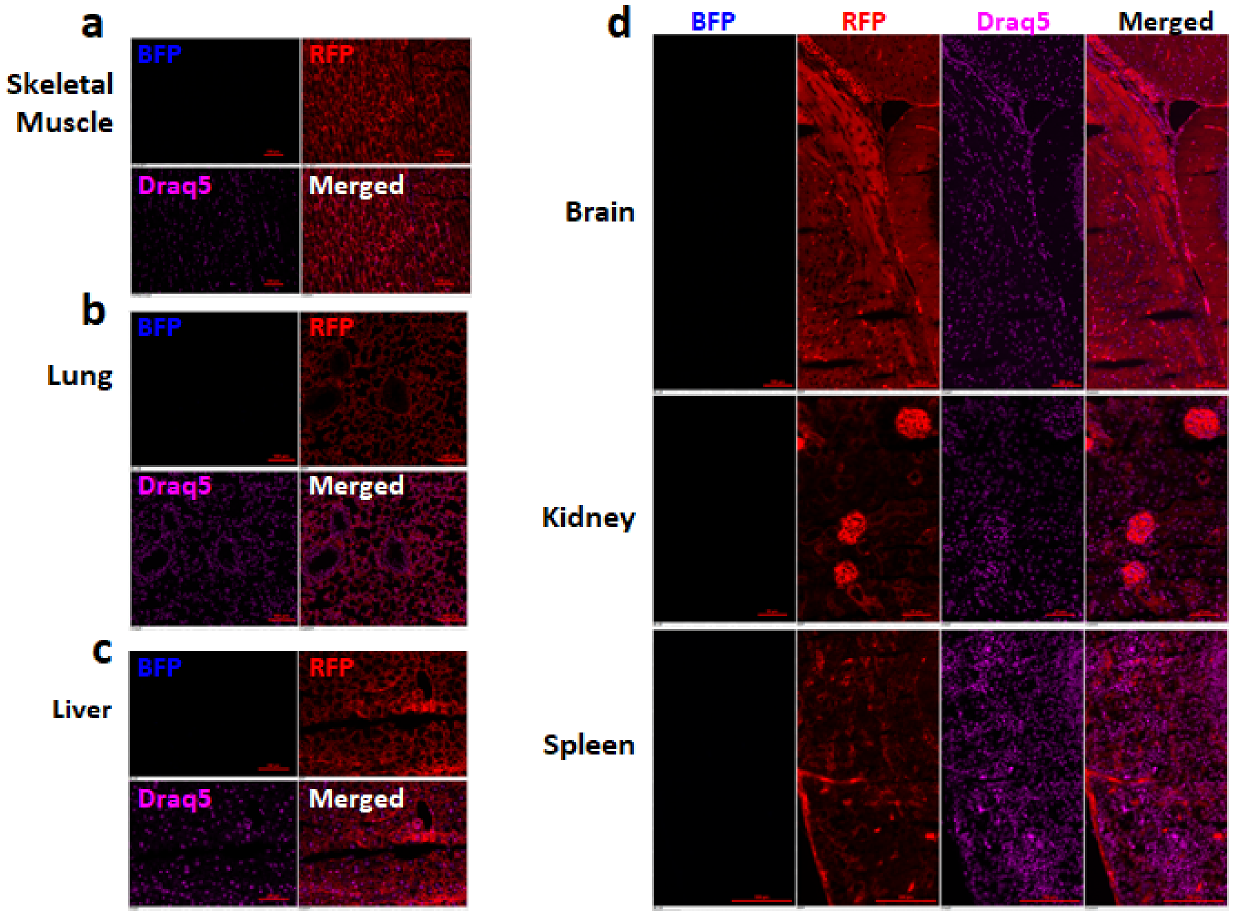

Supplementary Fig. S7. Absence of BFP signal in non-cardiac organs of double reporter BFP;RFPfl/GFP mice. No expression of BFP in skeletal muscle (**a**), lung (**b**), liver (**c**), brain, kidney or spleen (**d**). Both BFP and RFP are native fluorescent signals. Nuclei were stained with Draq5 (magenta). RFP signal expresses in all cells. Scale bars, 100 µm.

Supplementary Fig. S8

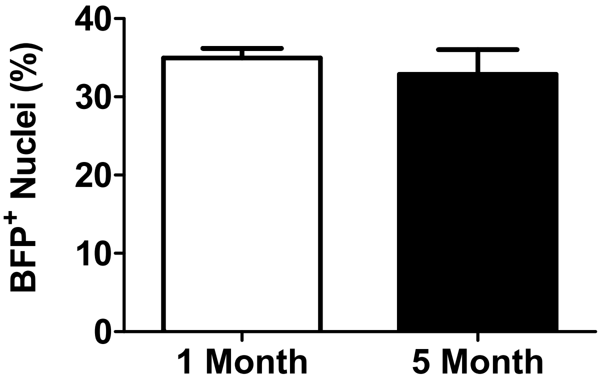

Supplementary Fig. S8. Comparable level of BFP myocyte nuclei in young and adult ventricle. There is no significant difference ($p>0.05$) of BFP^+ population in total ventricular nuclei in young (1 month; n=3) compared to adult (5 month; n=3) transgenic hearts evaluated by flow cytometry analysis on the nuclei from whole ventricles.

Supplementary Fig. S9

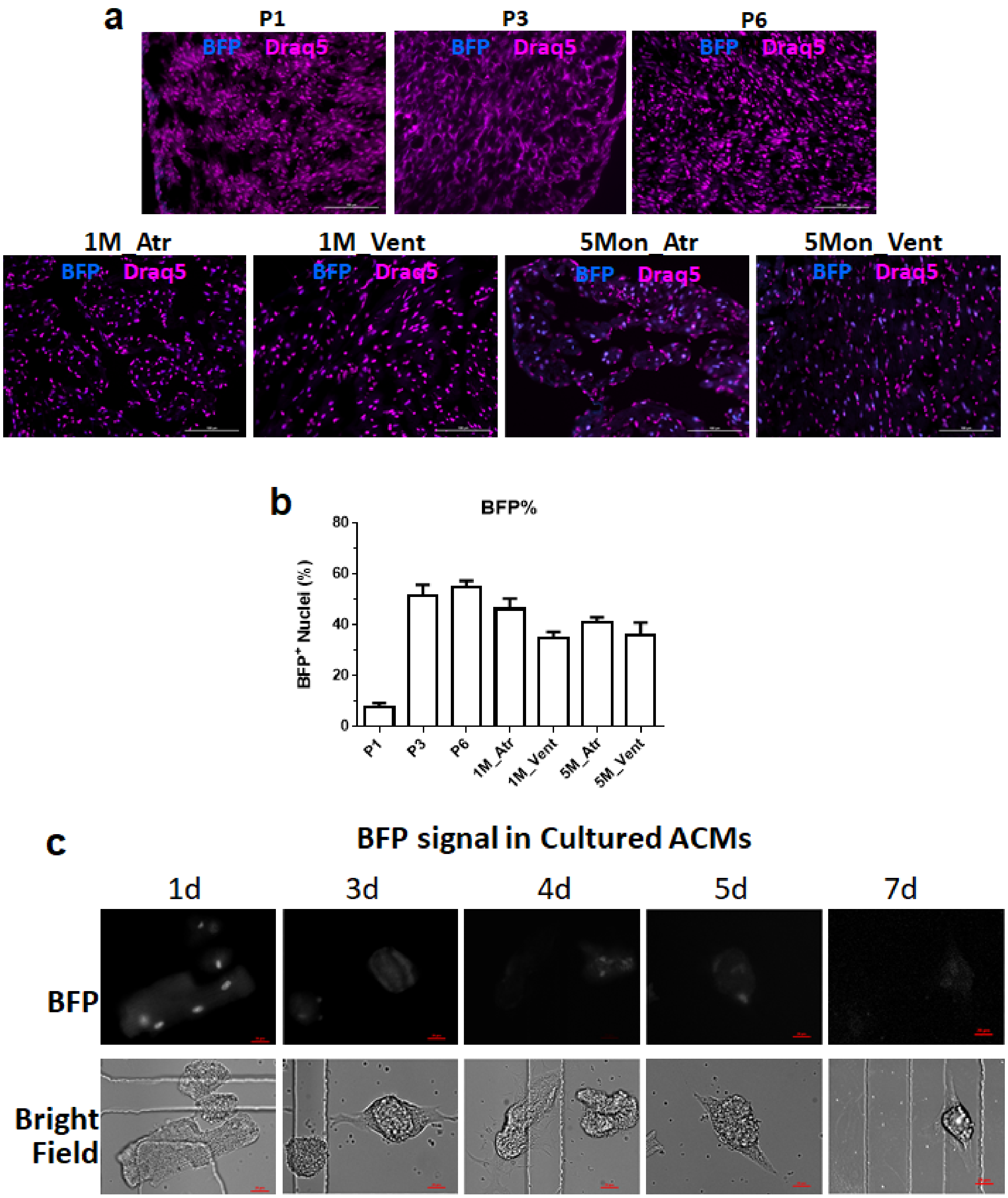

Supplementary Fig. S9. BFP Signal in transgenic BFP mouse hearts and dedifferentiated ACMs. a, Example microscope images showing native BFP signal (blue) in ventricles of transgenic mice at the age of postnatal 1 day (P1), 3 days (P3), 6 days (P6), and atrium (Atr) or ventricles (Vent) of 1 month or 6 months old hearts. Nuclei were stained by Draq5 (magenta).

Scale bar=100μm. **b,** Percentage of BFP^+ nuclei in mouse hearts during development *(left)* and during dedifferentiation cell culture *(right)*. One-way anova test $p<0.0001$. n=3 , 4, 3, 3, and 4 mice for P1, P3, P6, 1 month, and 5 month ages, respectively. **c,** Sample images of ACMs cultured in dedifferentiation condition.

Supplementary Fig. S10

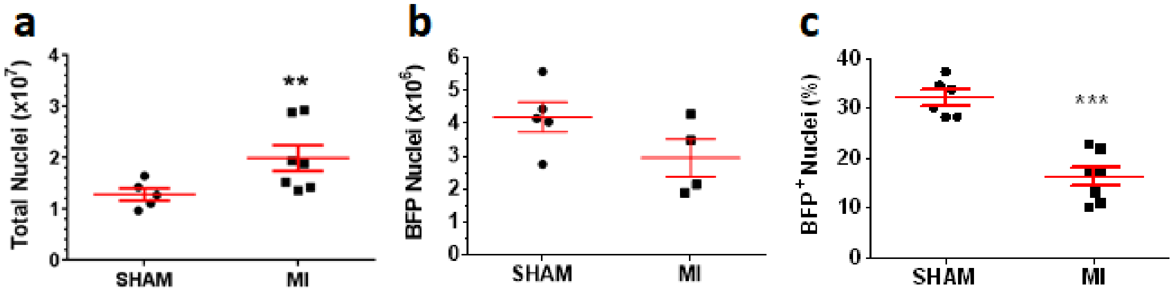

Supplementary Fig. S10. Reduction of BFP^{+} cardiac nuclei in post-infarct hearts. Three-week post-myocardial infarction (MI) or sham operation, total nuclei prepared from whole ventricles were measured by hemocytometer (**a**), and absolute BFP^{+} nuclei number and percentages were compared (**b**, **c**) based on flow cytometry and hemocytometer counts. Nuclei were identified by Draq5 staining. Data points represent individual animals. ** $p<0.005$, *** $p<0.001$; $p=0.1239$ for absolute BFP nuclei number in MI vs Sham (**b**).

Supplementary Fig. S11

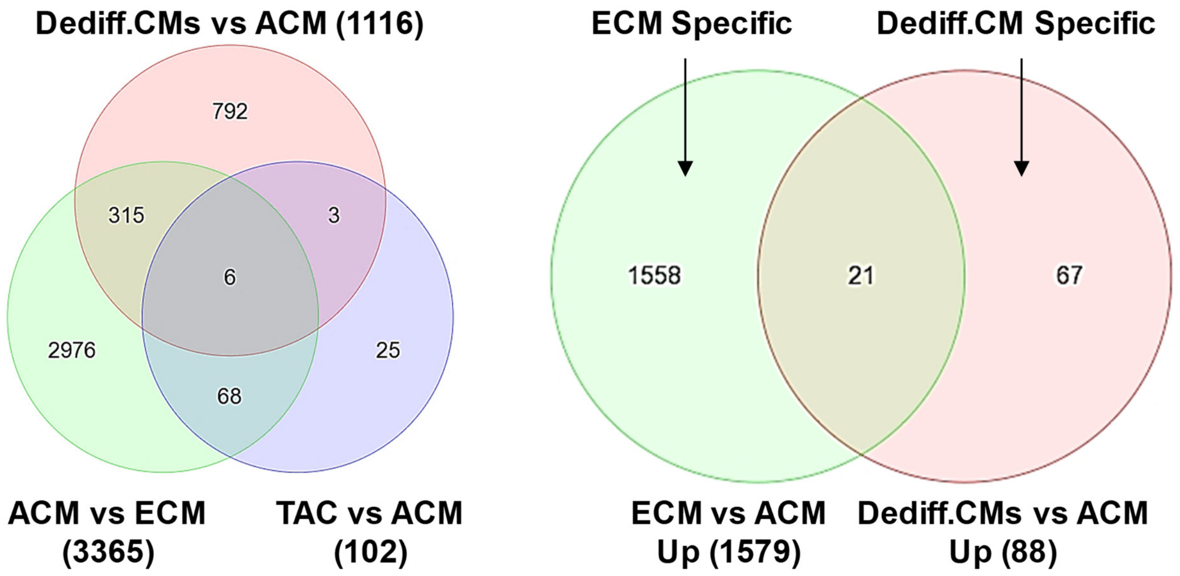

Supplementary Fig. S11. Whole-Transcriptomes analysis of cultured dedifferentiated cardiomyocytes (Dediff.CMs), adult cardiomyocytes (ACM), embryonic cardiomyocytes (ECM), and hypertrophic cardiomyocytes (TAC) based on microarray data. Numbers of differentially expressed genes are indicated in Venn diagrams.

Fig. S12.

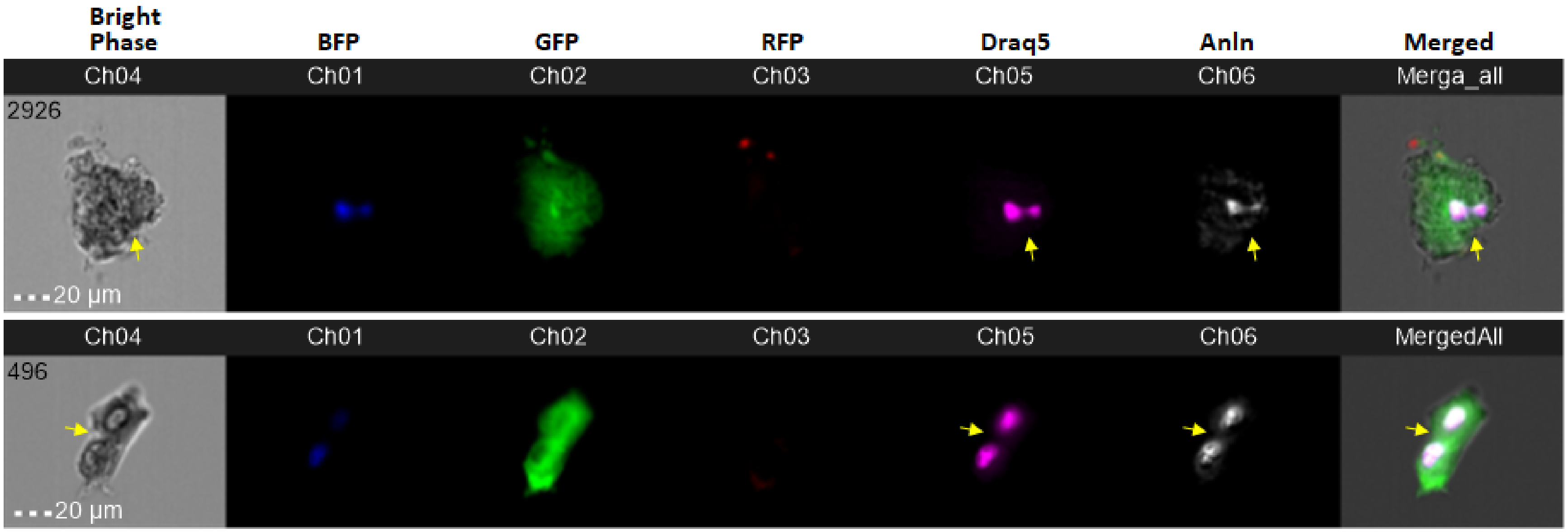

Supplementary Fig. S12. Cytokinesis in dedifferentiated pre-existing cardiomyocytes. ImageStream analysis of Anillin (Anln)$^{+}$ mitotic cardiomyocytes. Nuclei (stained by Draq5) and Anln (revealed by APC-Cy7-conjugated antibody), and bright phase image, BFP, GFP, RFP reporters are shown in Ch05, Ch06, Ch04, Ch01, Ch02, and Ch03, respectively. Yellow arrows denote the mitotic mid-body plane or the segregation furrow.

Fig. S13.

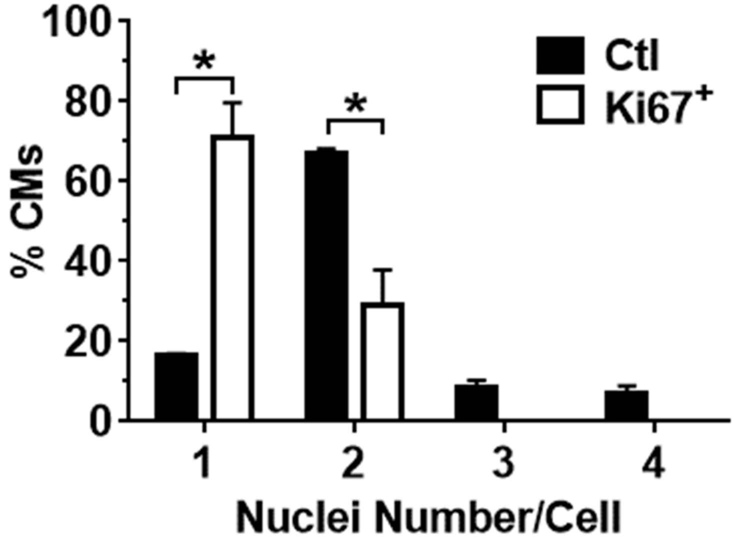

Supplementary Fig. S13. Number of nuclei in normal (Ctl) or Ki67^{+} cardiomyocytes. n= 3 mice for Ctl, and n=4 post-infarct mice for Ki67^{+}. $p<0.0001$ in 2-way ANOVA test. * $p<0.05$ post-hoc Sidak test.

Fig. S14.

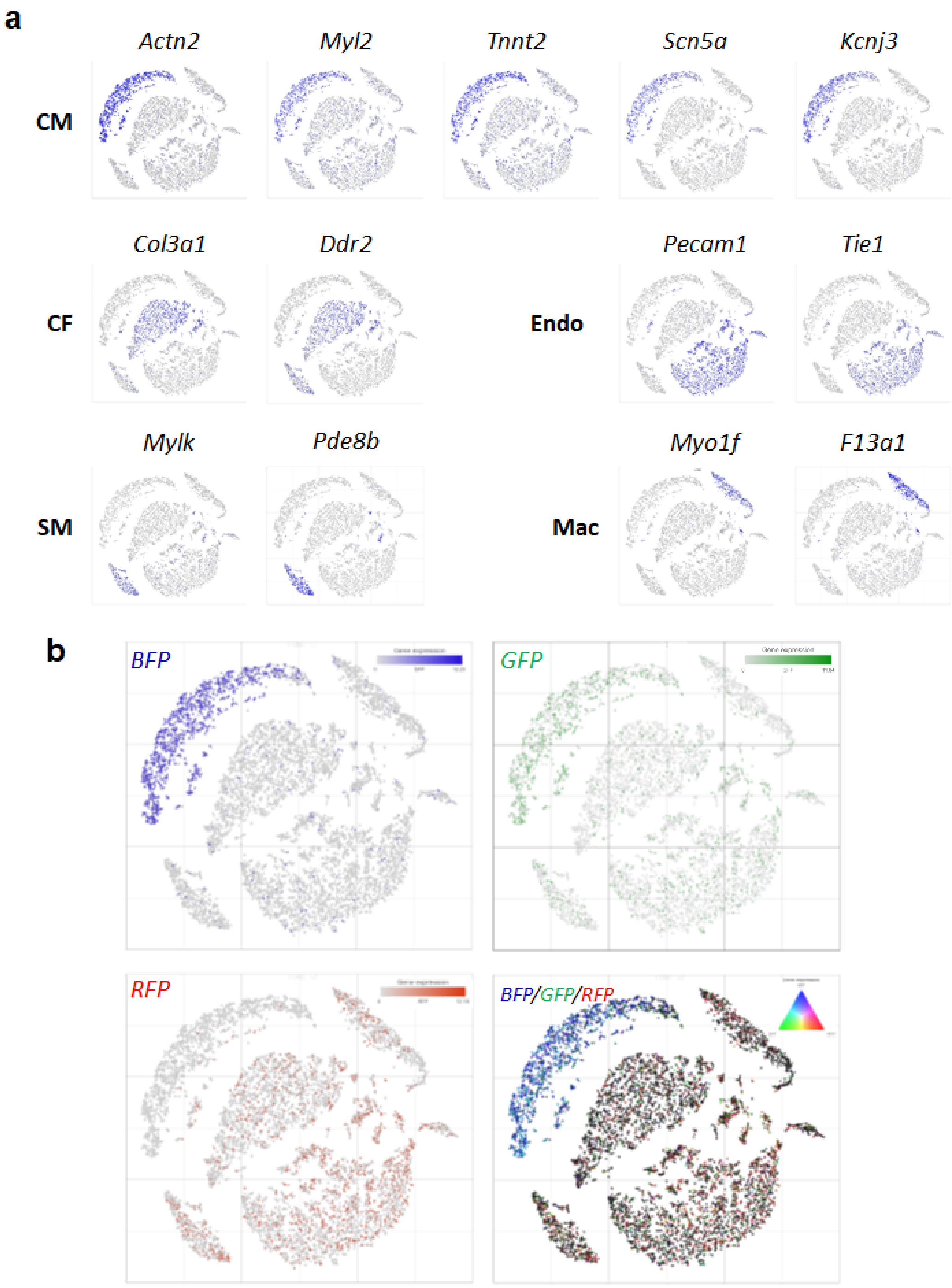
a
Actn2
Myl2
Tnnt2
Scn5a
Kcnj3
CM
Col3a1
Ddr2
Pecam1
Tie1
CF
Endo
Mylk
Pde8b
Myo1f
F13a1
SM
Mac
b
BFP
GFP
RFP
BFP/GFP/RFP

Supplementary Fig. S14. Single-nucleus RNA-seq reveals heart cell identities. (a) T-SNE plots as for the control heart in Fig. 7a, showing positive expression level (low-grey; high-blue) of markers in cardiomyocytes (CM), cardiac fibroblasts (CF), endothelial cells (Endo), smooth muscle cells (SM), and macrophages (Mac). (**b**) Expression of *BFP*, *GFP*, and *RFP* reporter genes in control cardiac nuclei.

Fig. S15

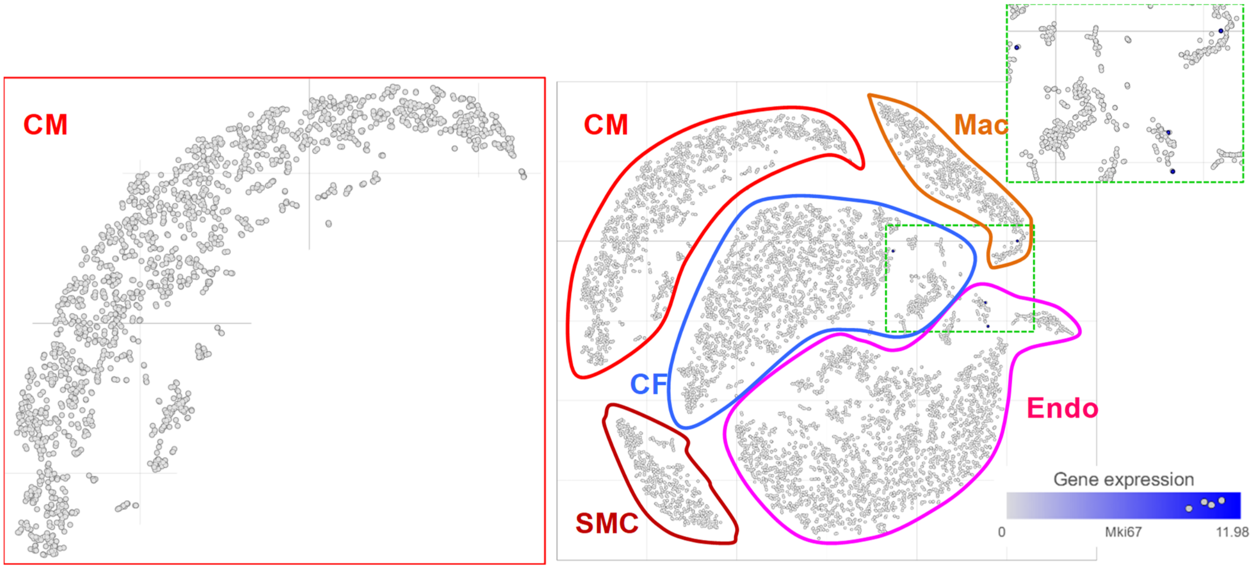

Supplementary Fig. S15. t-SNE plot for nuclei from control heart showing nuclei the expression of $GFP^{+}BFP^{-}RFP^{-}Ki67^{+}$ in normal control heart. The 4 cycling nuclei (inside the green-box) were not among the myocyte cluster. *Ki67* expression level: low-grey; high-blue. CMs: cardiomyocytes; CF: cardiac fibroblasts; Endo: endothelial cells; Mac: Macrophages; SMC: smooth muscle cells.

Fig. S16.

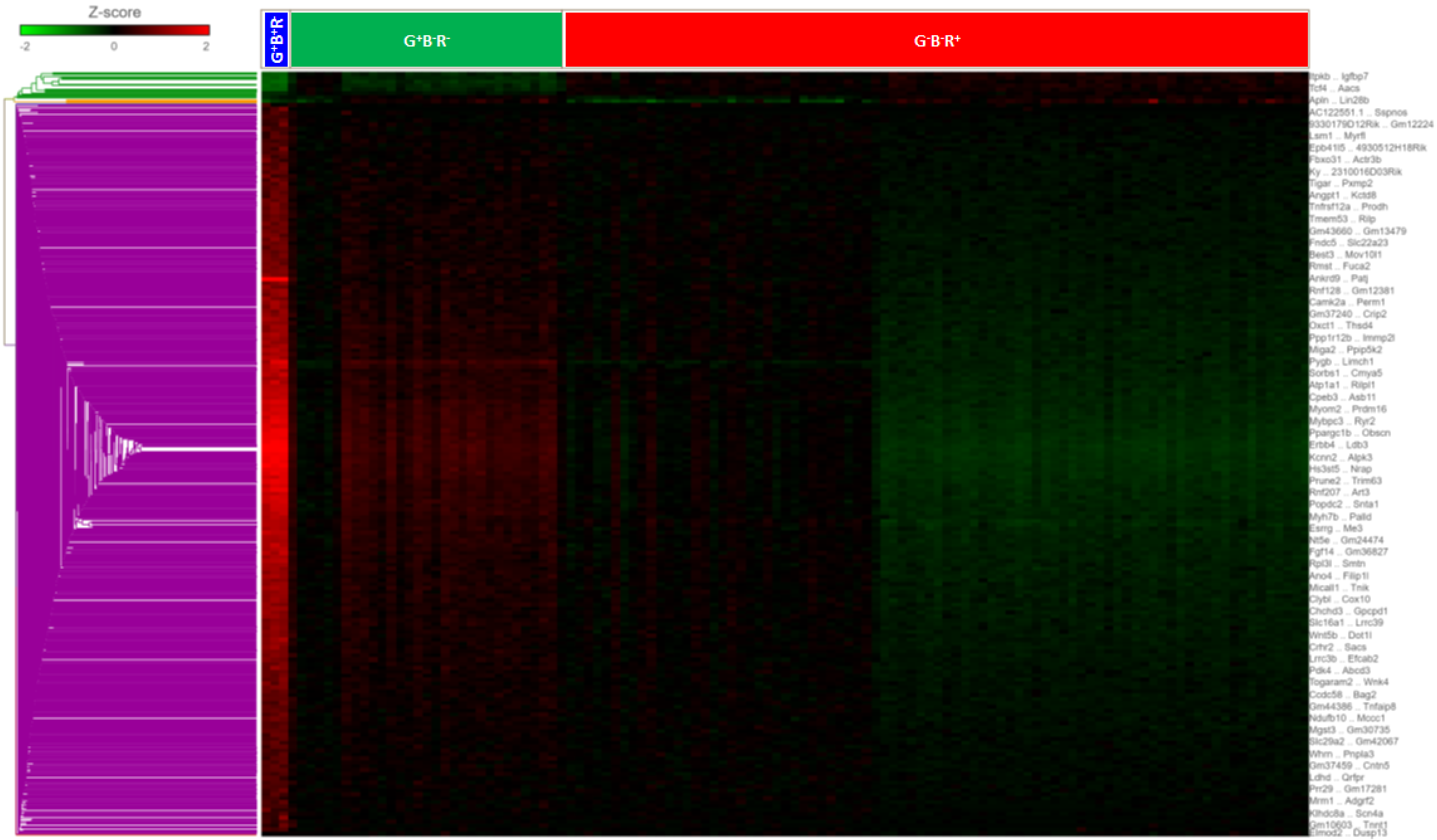

Supplementary Fig. S16. Hierarchical clustering of the DEGs (FDR-adjusted p≤1e-8, |fold-change|>2) between *GFP*$^{+}$*BFP*$^{-}$*RFP*$^{-}$ (*G*$^{+}$*B*$^{-}$*R*$^{-}$) and *GFP*$^{+}$*BFP*$^{+}$ *RFP*$^{-}$ (*G*$^{+}$*B*$^{+}$*R*$^{-}$) nuclei from post-infarct hearts.

Fig. S17a.

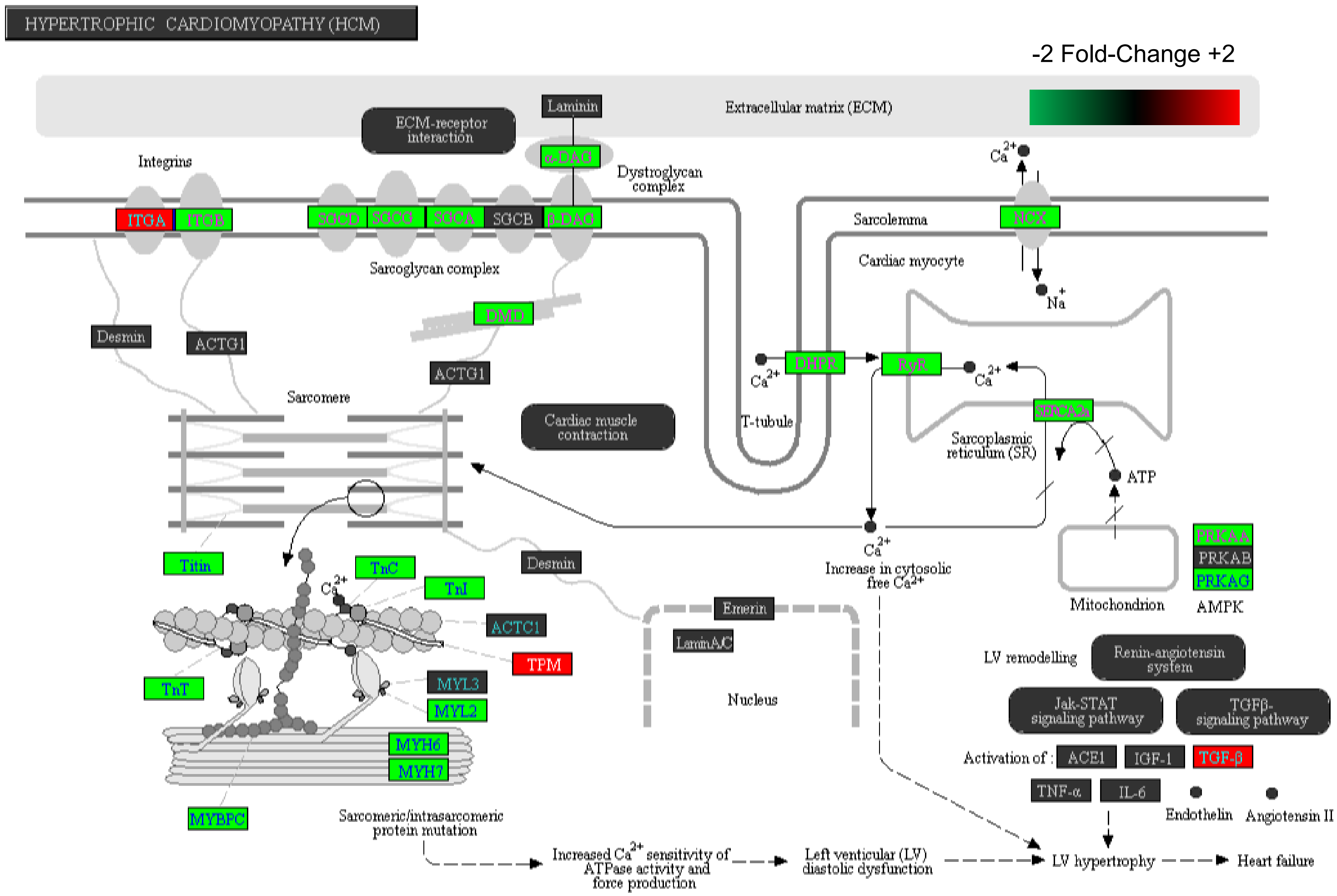

Supplementary Fig. S17. Enrichment of signaling pathways by the by the DEGs (|Fold-change|>2, FDR p≤0.01) between BFP$^-$ and BFP$^+$ CM nuclei (GFP$^+$RFP$^-$) from post-infarct hearts. a, *Hypertrophic Cardiomyopathy (HCM)* pathway (Enrichment Score 34.6911, p 8.59E-16).

Fig. S17b

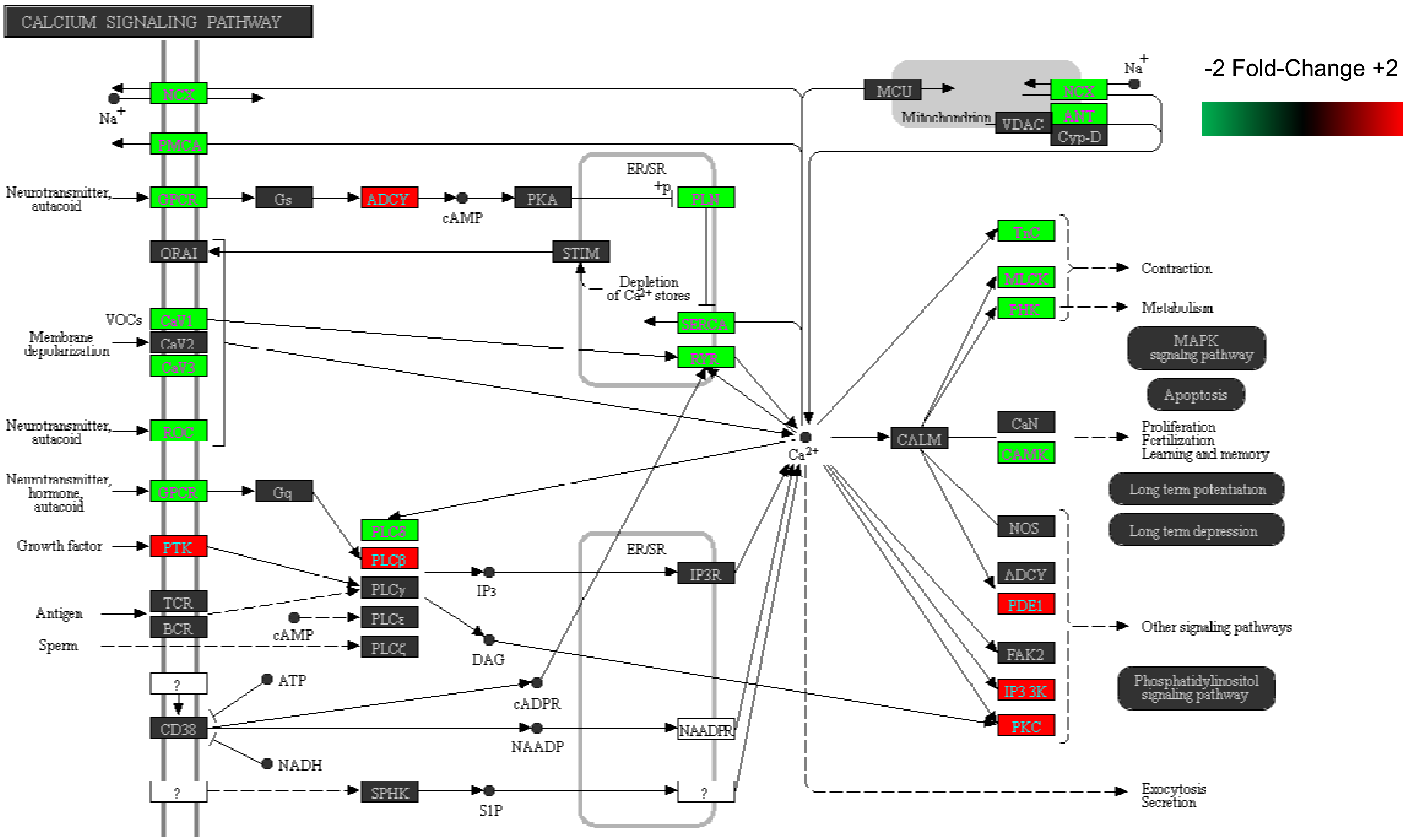

Supplementary Fig. S17. Enrichment of signaling pathways by the by the DEGs (|Fold-change|>2, FDR p≤0.01) between BFP$^-$ and BFP$^+$ CM nuclei (GFP$^+$RFP$^-$) from post-infarct hearts. b, *Calcium signaling pathway* (Enrichment Score 24.0338, p 3.65E-11).

Fig. S17c

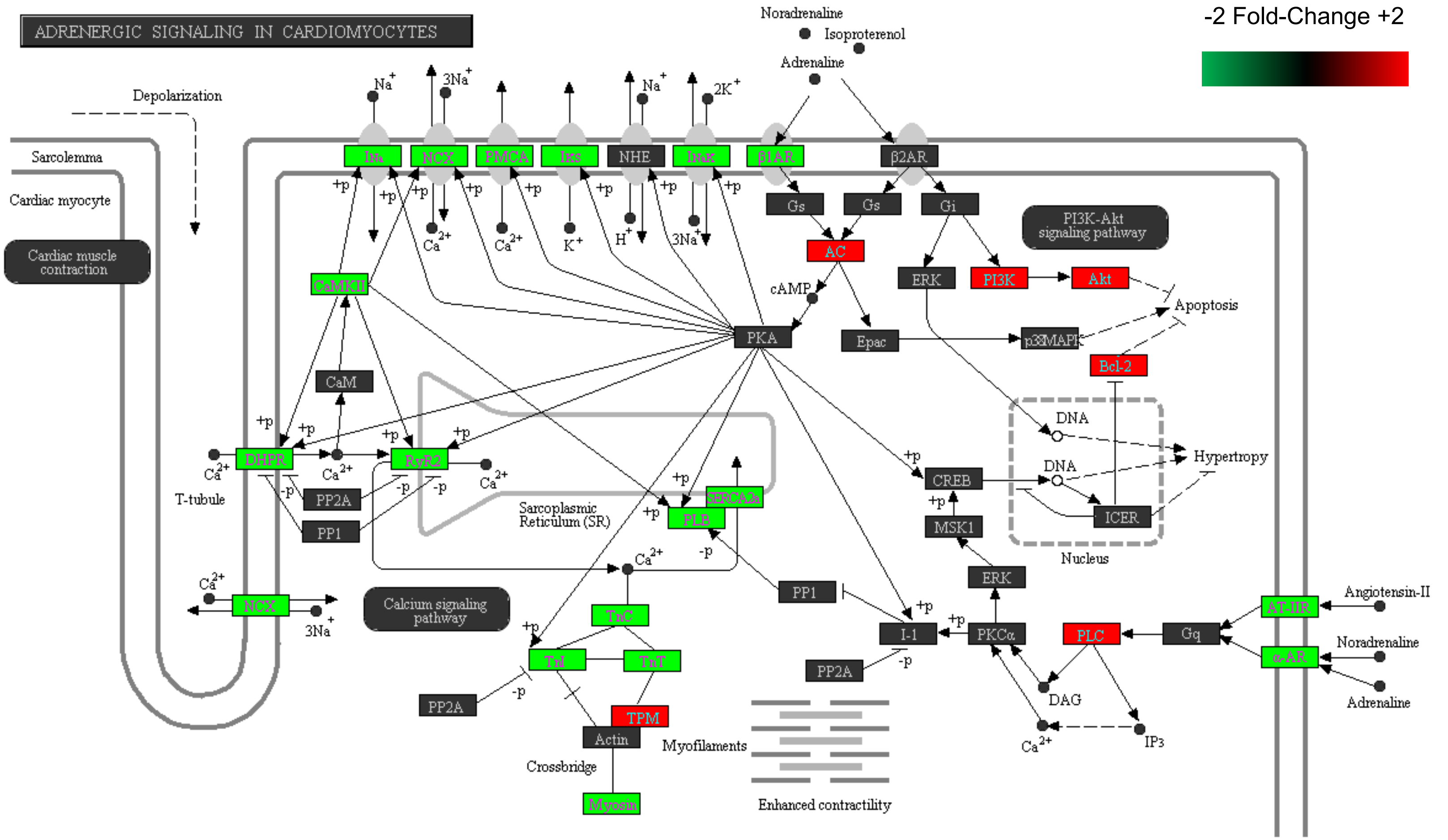

Supplementary Fig. S17. **Enrichment of signaling pathways by the by the DEGs (|Fold-change|>2, FDR p≤0.01) between BFP$^-$ and BFP$^+$ CM nuclei (GFP$^+$RFP$^-$) from post-infarct hearts. c**, *Adrenergic signaling in cardiomyocytes* signaling pathway (Enrichment score 23.917p 4.10E-11).

Fig. S17d

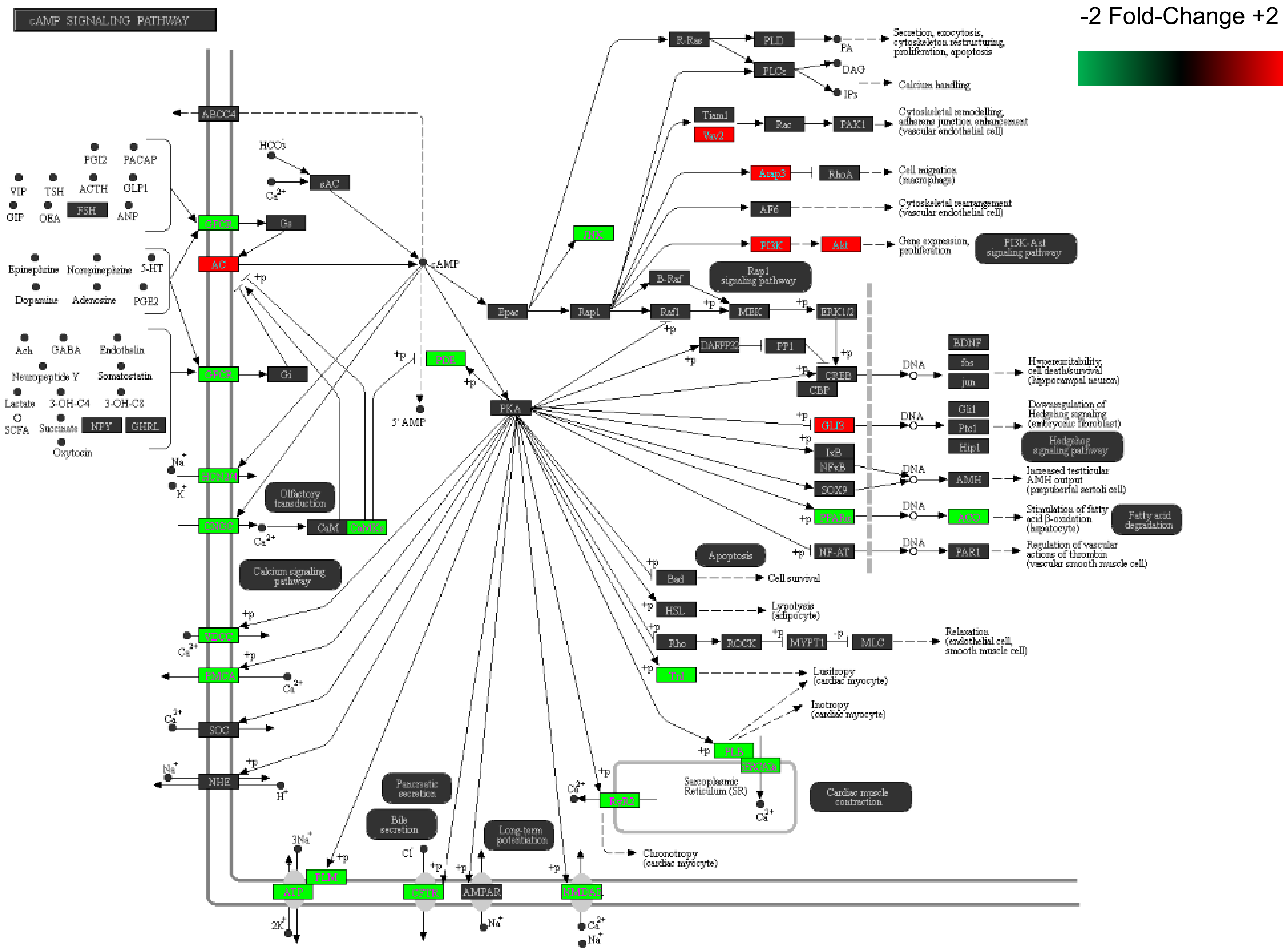

Supplementary Fig. S17. **Enrichment of signaling pathways by the by the DEGs (|Fold-change|>2, FDR p≤0.01) between BFP$^-$ and BFP$^+$ CM nuclei (GFP$^+$RFP$^-$) from post-infarct hearts. d**, *cAMP signaling pathway* (Enrichment Score 18.5731, p 8.59E-09).

Fig. S17e

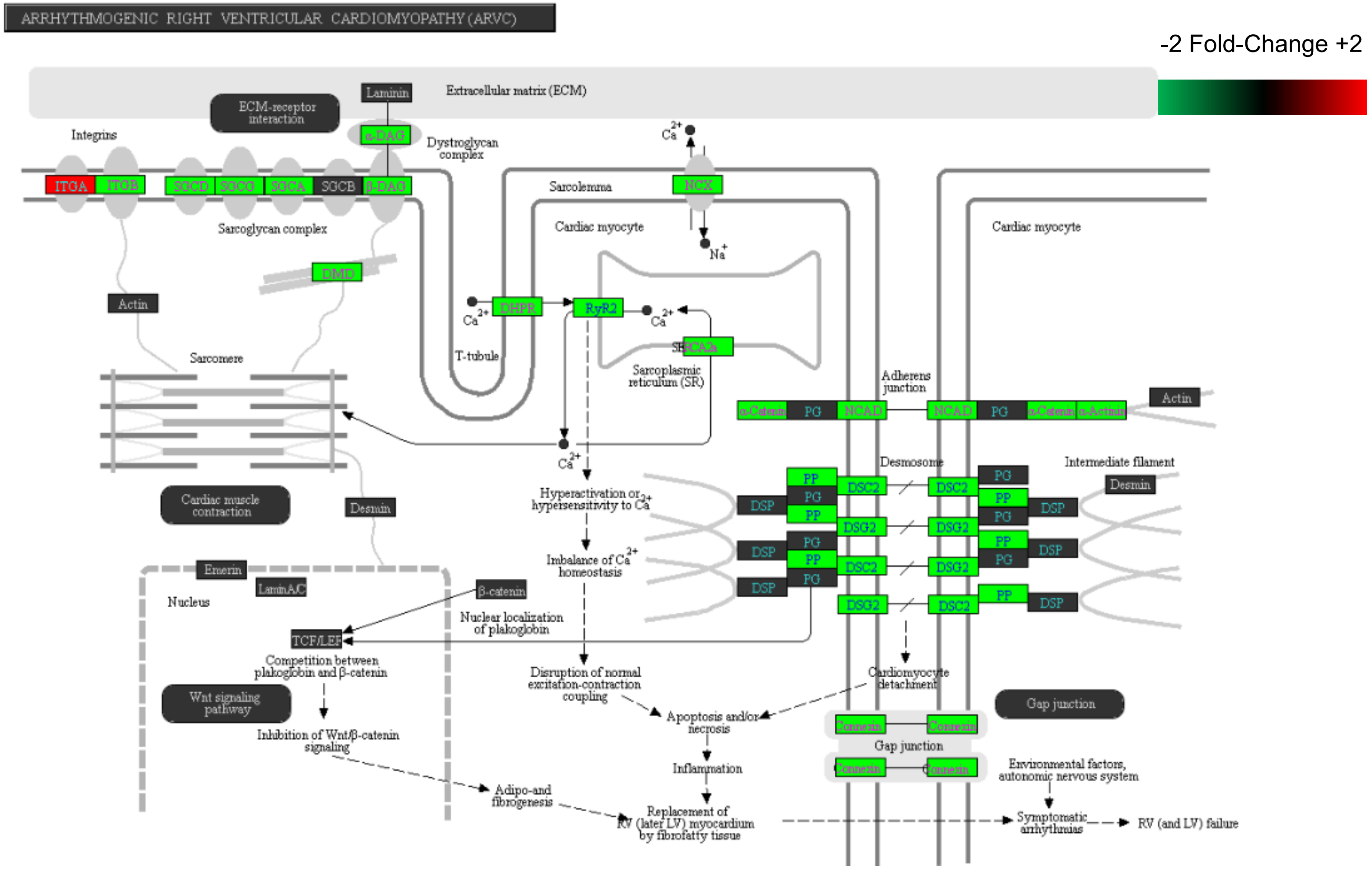

Supplementary Fig. S17. **Enrichment of signaling pathways by the by the DEGs (|Fold-change|>2, FDR p≤0.01) between BFP⁻ and BFP⁺ CM nuclei (GFP⁺RFP⁻) from post-infarct hearts. e**, *Arrhythmogenic right ventricular cardiomyopathy* (ARVC) (Enrichment Score 23.547, p 5.94E-11).

Fig. S17f.

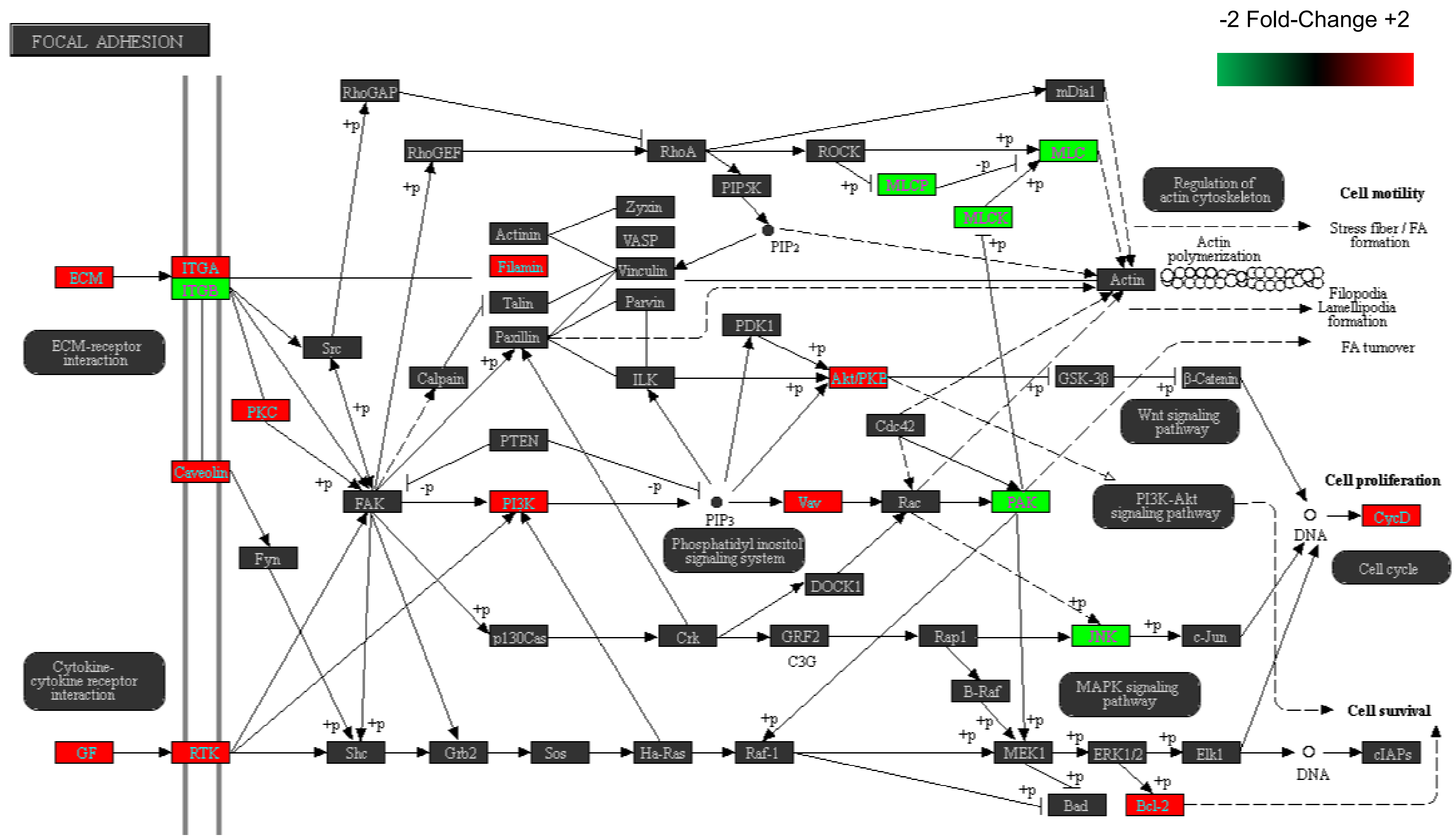

Supplementary Fig. S17. **Enrichment of signaling pathways by the by the DEGs (|Fold-change|>2, FDR p≤0.01) between BFP⁻ and BFP⁺ CM nuclei (GFP⁺RFP⁻) from post-infarct hearts. f,** *Focal adhesion* pathway (Enrichment Score 16.5489, p 6.50E-08).

Fig. S17g.

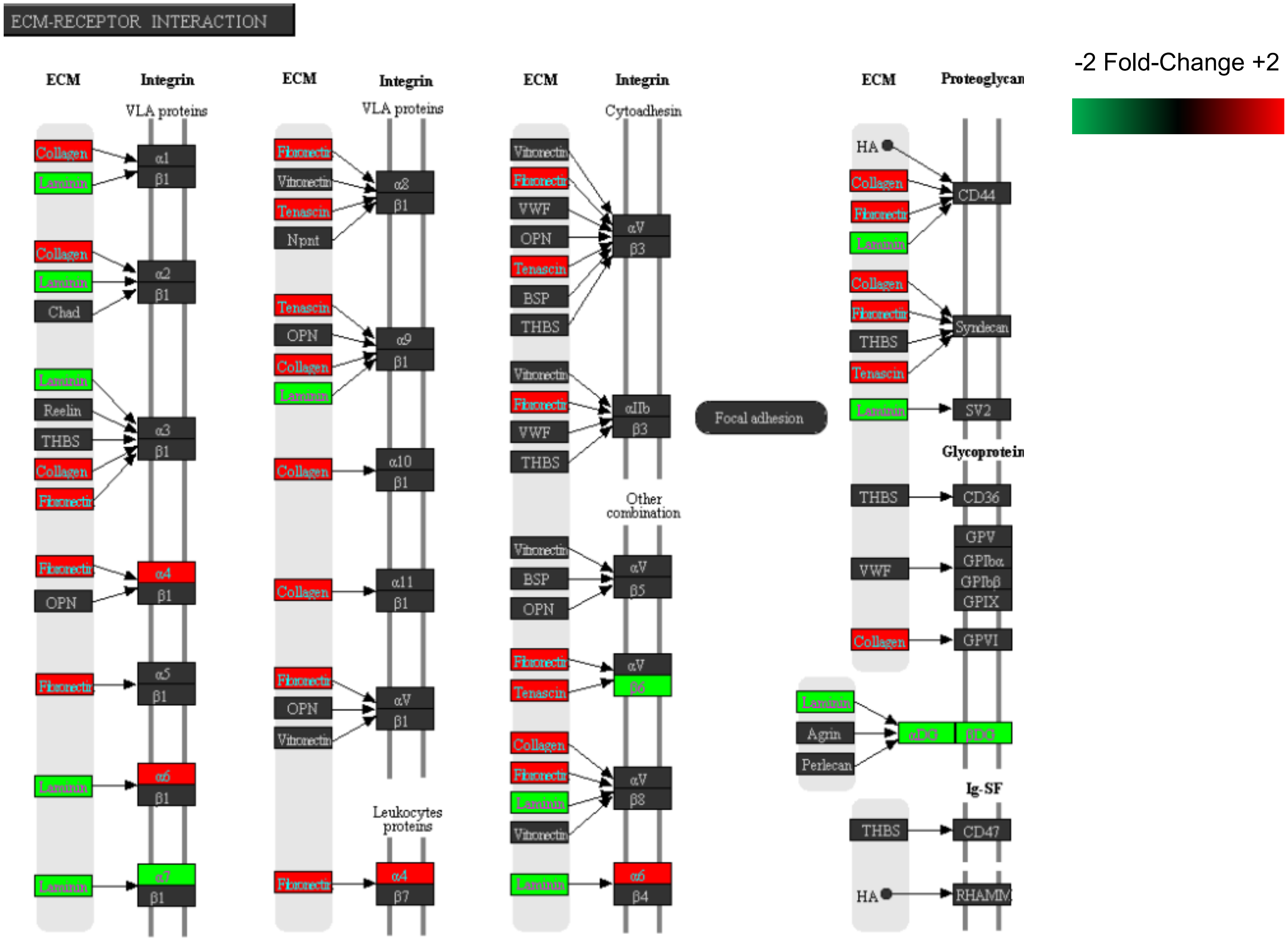

Supplementary Fig. S17. Enrichment of signaling pathways by the by the DEGs (|Fold-change|>2, FDR p≤0.01) between BFP$^-$ and BFP$^+$ CM nuclei (GFP$^+$RFP$^-$) from post-infarct hearts. g, *ECM-receptor interaction* pathway (Enrichment Score 5.75899, p 0.0031543).

Fig. S17h.

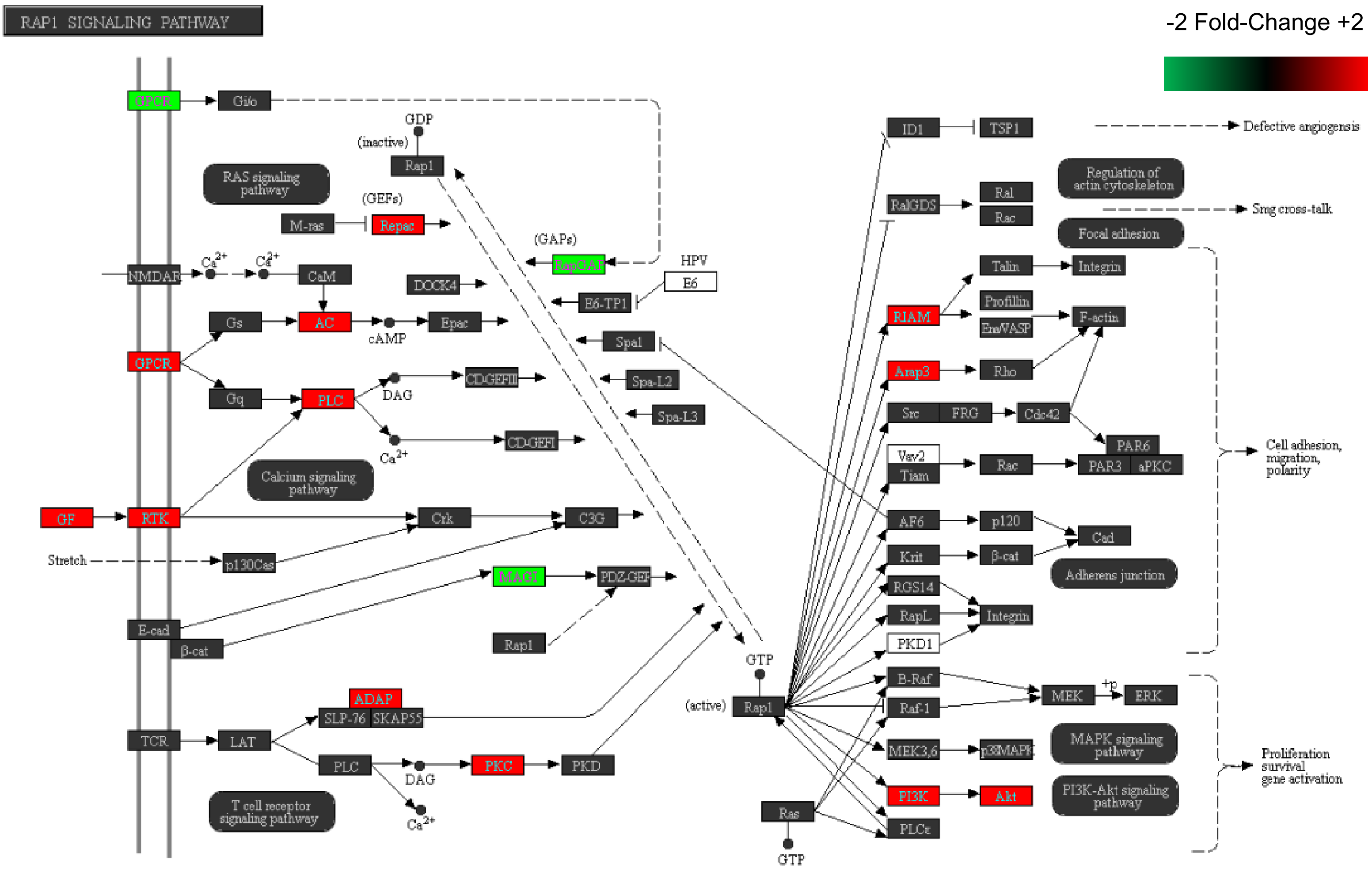

Supplementary Fig. S17. **Enrichment of signaling pathways by the by the DEGs (|Fold-change|>2, FDR p≤0.01) between BFP^{-} and BFP^{+} CM nuclei (GFP^{+}RFP^{-}) from post-infarct hearts. h**, *Rap1 signaling pathway* (Enrichment Score 7.29744, p 0.000677271).

Fig. S17i.

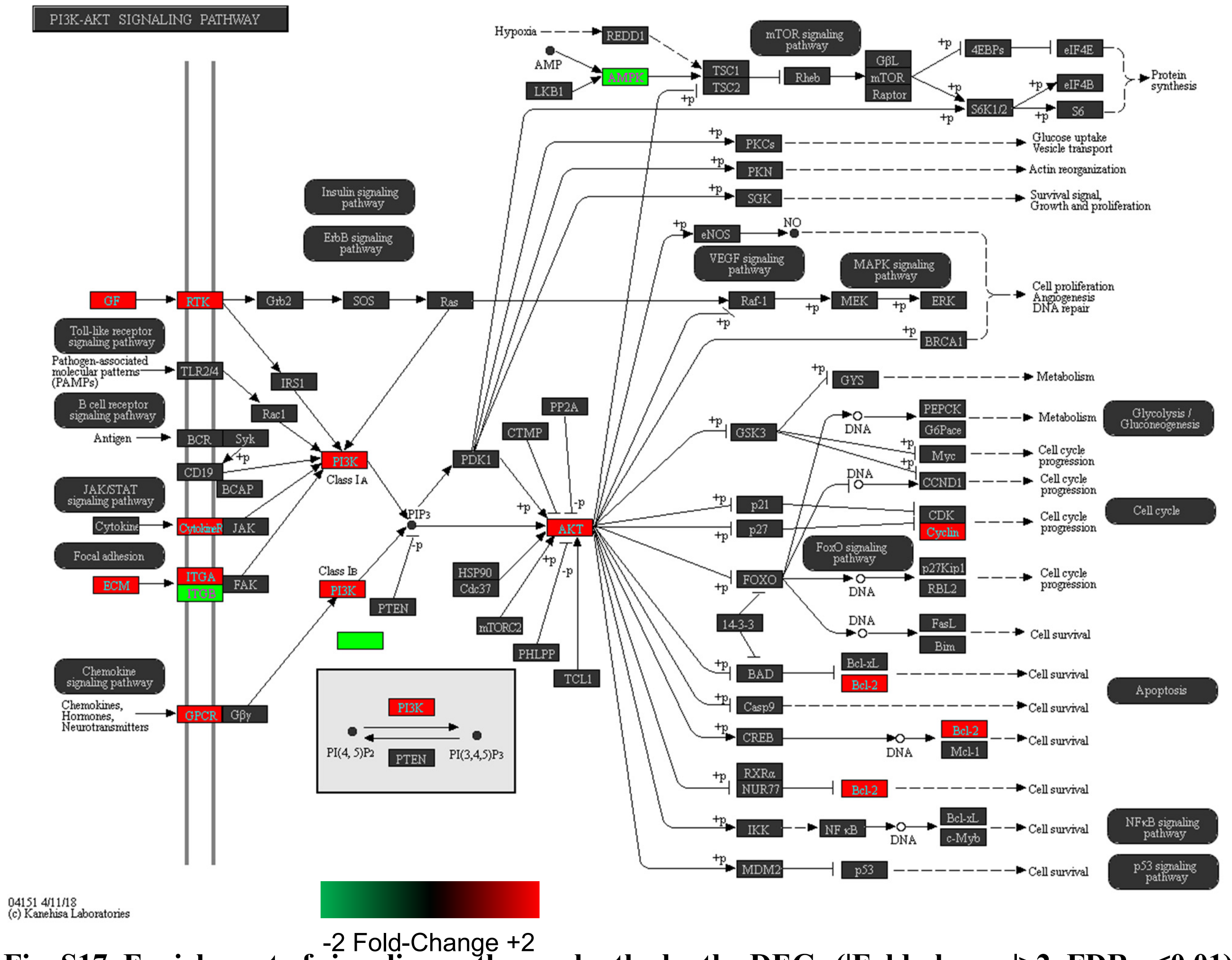

Supplementary Fig. S17. **Enrichment of signaling pathways by the by the DEGs (|Fold-change|>2, FDR p≤0.01) between BFP$^-$ and BFP$^+$ CM nuclei (GFP$^+$RFP$^-$) from post-infarct hearts. i,** *PI3K-Akt signaling pathway* (Enrichment Score 8.78997, p 0.000152253).

Fig. S18.

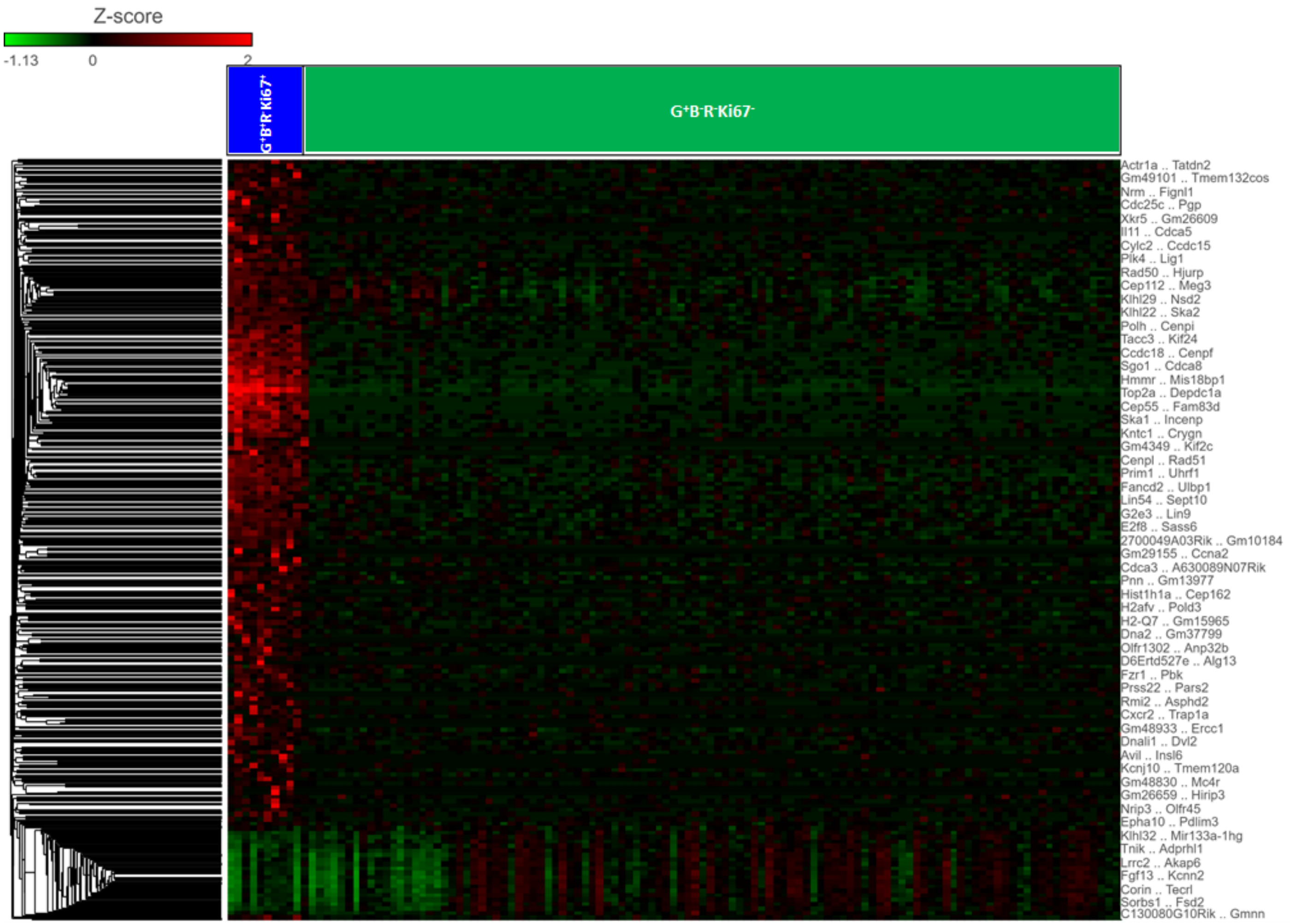

Supplementary Fig. S18. Hierarchical clustering of the DEGs (FDR-adjusted p≤1E-8, |fold-change|>2) between *Mki67*⁺ and *Mki67*⁻ dedifferentiated cardiomyocyte (*GFP*⁺*BFP*⁻ *RFP*⁻) nuclei from post-infarct hearts.

Fig. S19.

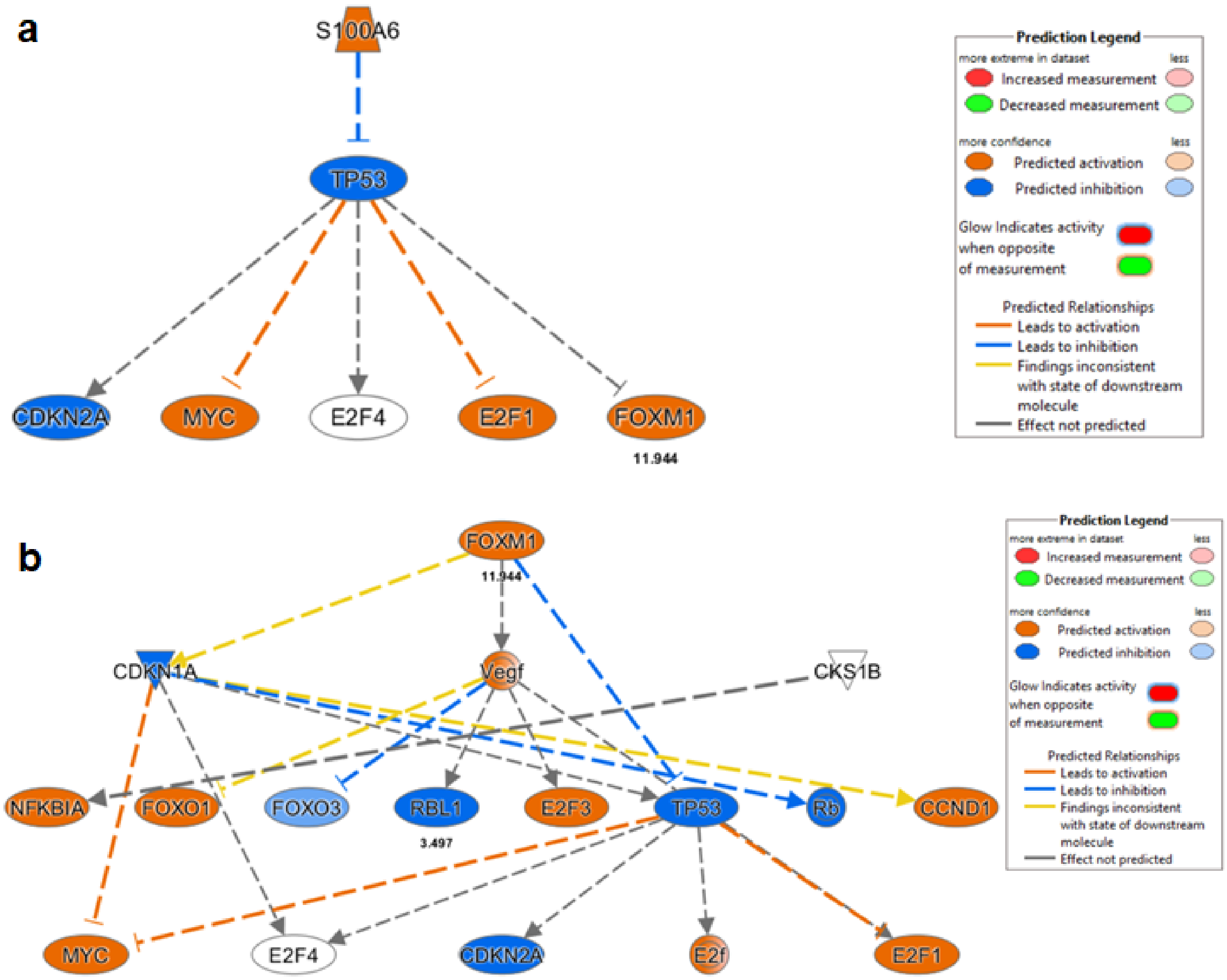

Supplementary Fig. S19. Upstream transcription regulator analysis predicts that S100A6 (**a**; Activation Z-score 3.162, with a *p* value of overlap 1.54E-9) and FOXM1 (**b**; Activation Z-score 5.101, with a *p* value of overlap 4.01E-21) are putative regulators leading to the gene expression reprogramming in dedifferentiated cycling cardiomyocytes (*GFP*$^{+}$*BFP*$^{-}$*RFP*$^{-}$*Ki67*$^{+}$). Fold changes of gene expression in *GFP*$^{+}$*BFP*$^{-}$*RFP*$^{-}$*Ki67*$^{+}$ nuclei compared to *GFP*$^{+}$*BFP*$^{-}$*RFP*$^{-}$*Ki67*$^{-}$ nuclei are noted under related genes.

C. Supplementary Tables

Supplementary Table S1. List of genes specific to dedifferentiated cardiomyocytes. Also refer to Supplementary Fig. S11.

Please see separated Excel spreadsheet file.

Supplementary Table S2. Top group biomarkers of graph-clustered nuclei in the control heart. Top 15 differentially expressed genes in each graph cluster shown in Fig. 7a.

Please see separated Excel spreadsheet file.

Supplementary Table S3. Top group biomarkers of graph-clustered cells in post-infarct hearts. Top 15 differentially expressed genes in each graph cluster shown in Fig. 7a.

Please see separated Excel spreadsheet file.

Supplementary Table S4. Cell populations identified by unsupervised graph-based clustering on single-nuclear transcriptomes.

Cell Types (%)	Control	MI
**Cardiomyocytes**	21.0%	15.0%
**Fibroblasts**	27.9%	25.8%
**Endothelial Cells**	35.4%	23.7%
**Smooth muscle cells**	6.8%	5.2%
**Macrophages/Immuno cells**	9.0%	24.6%
**Macrophages/Endothelial cells?**	/	5.9%
**Total**	100%	100%

8550 nuclei for the Control group, and 22,992 nuclei for the infarct (MI) group.

Supplementary Table S5. Pathway enrichment analysis on DEGs (FDR-adjusted p≤0.01, |fold-change|>2) of dedifferentiated myocyte nuclei (*BFP^- vs. BFP^+*, in *GFP^+RFP^-* ACM population) in post-infarct hearts.

Please see separated Excel spreadsheet file.

Supplementary Table S6. Pathway Enrichment analysis on DEGs (FDR-adjusted p≤0.01, |fold-change|>2) of dedifferentiated cycling myocytes (*$GFP^+BFP^-RFP^-Ki67^+$ vs. $GFP^+BFP^-RFP^-Ki67^-$*) in post-infarct hearts.

Please see separated Excel spreadsheet file.

Supplementary Table S7. Gene Set Enrichment analysis on DEGs (FDR-adjusted p≤0.01, |fold-change|>2) of dedifferentiated cycling myocytes (*$GFP^+BFP^-RFP^-Ki67^+$ vs. $GFP^+BFP^-RFP^-Ki67^+$*) in post-infarct hearts.

Please see separated Excel spreadsheet file.

Supplementary Table S8. RT-qPCR primers for gene expression analysis.

Gene	Forward Primer (5'… 3')	Reverse Primer (5'… 3')
Ccnb1	ACGGGCATTCCTCTTCCCCT	TACCACAGGGTCACCACCTCA
Cdk2	ACGGGCATTCCTCTTCCCCT	TACCACAGGGTCACCACCTCA
Fosl1	GGTGCAAGTGGTTCAGCCCAAG	CAAGGTGGAACTTCTGCTGCTGG
Gapdh	CCAATGTGTCCGTCGTGGATCT	GTTGAAGTCGCAGGAGACAACC
Myc	GGTCCGACTCGCCTCACTCA	TTCCAACCGTCCGCTCACTC
Myh6	AGAAGCCCAGCGCTCCCTCA	GGGCGTTCTTGGCCTTGCCT
Myh7	GCGACTCAAAAAGAAGGACTTTG	GGCTTGCTCATCCTCAATCC
S100a6	GGGCCCGCTAAACTCCCTC	GGCTAGAAGAAGCGCACGGTC
Sox4	TGGGGTGATGCGTTTGGCATT	TCCACGAGGTCCCCATGTCC
Thbs1	CCTGCCCACCACGATTCACT	TCGCGGTTTGCACACCTGTT
Tmsb10	AATCTTCTGGGGCTAGCAGGC	CTTCCGCGAACGCCTCTCAA
